# Random Mutagenesis Identifies a C-Terminal Region of YopD Important for *Yersinia* Type III Secretion Function

**DOI:** 10.1371/journal.pone.0120471

**Published:** 2015-03-25

**Authors:** Rebecca Solomon, Weibing Zhang, Grace McCrann, James B. Bliska, Gloria I. Viboud

**Affiliations:** 1 Clinical Laboratory Science, School of Health, Technology and Management, Stony Brook University, Stony Brook, New York, United States of America; 2 Department of Molecular Genetics and Microbiology, Center for Infectious Diseases, School of Medicine, Stony Brook University, Stony Brook, New York, United States of America; Indian Institute of Science, INDIA

## Abstract

A common virulence mechanism among bacterial pathogens is the use of specialized secretion systems that deliver virulence proteins through a translocation channel inserted in the host cell membrane. During *Yersinia* infection, the host recognizes the type III secretion system mounting a pro-inflammatory response. However, soon after they are translocated, the effectors efficiently counteract that response. In this study we sought to identify YopD residues responsible for type III secretion system function. Through random mutagenesis, we identified eight *Y*. *pseudotuberculosis* yopD mutants with single amino acid changes affecting various type III secretion functions. Three severely defective mutants had substitutions in residues encompassing a 35 amino acid region (residues 168–203) located between the transmembrane domain and the C-terminal putative coiled-coil region of YopD. These mutations did not affect regulation of the low calcium response or YopB-YopD interaction but markedly inhibited MAPK and NFκB activation. When some of these mutations were introduced into the native *yopD* gene, defects in effector translocation and pore formation were also observed. We conclude that this newly identified region is important for YopD translocon function. The role of this domain *in vivo* remains elusive, as amino acid substitutions in that region did not significantly affect virulence of *Y*. *pseudotuberculosis* in orogastrically-infected mice.

## Introduction

Pathogenic *Yersinia* species require the function of a type III secretion system (T3SS) to successfully establish infection. This specialized secretion machinery delivers a series of effector proteins (YopE, H, J, K, M, O, T) into the host cell that target different cell signaling molecules to interfere with the host immune system [1, 2]. In the prevailing model, effectors are thought to travel through a needle-like conduit and to traverse the host cell membrane through a translocation channel formed by translocator proteins YopB and YopD [3–6]. Located at the tip of the needle is LcrV, a protein that interacts with YopB and YopD allegedly assisting in the assembly of the translocation channel prior to their insertion into the host cell membrane [7, 8].

Activation of the T3SS requires contact of the bacteria with the host cell. However, *in vitro*, Yop secretion can be stimulated by culturing bacteria at 37°C at low calcium concentration. This low calcium condition also results in growth arrest. Thus, wild type *Yersinia* is said to exhibit a calcium-dependent growth (CD) at 37°C. Under non-permissive high calcium conditions, YscM1/LcrQ in cooperation with YopD and its cytosolic chaperone SycD/LcrH) block *yop* mRNA translation [9–15]. Consequently, a *yopD* mutant produces Yops constitutively and has a temperature-sensitive (TS) growth phenotype irrespective of the calcium concentration. Two chaperon-binding sites have been reported for YopD, one spanning residues 53 to 149 and the other across residues 278 to 292 [16]. Deletion of any of those YopD regions results in a TS growth and deregulation of Yop synthesis [17].

Infection of cultured cells with *Yersinia* mutants lacking multiple effector Yops results in the formation of open pores in the plasma membrane and subsequent osmotic lysis [18]. This process is referred as “pore formation” and requires the function of YopB and YopD [18–20]. On the other hand, during infection with wild-type *Yersinia*, effectors YopE and YopT prevent pore formation by inhibiting Rho GTPases [20, 21]. These signaling molecules control a variety of cellular functions including regulation of the actin cytoskeleton. The first evidence that translocon insertion induces a host response was provided by data demonstrating YopB/D-dependent activation of Rho GTPases during *Yersinia* infection [20]. Interestingly, inhibition of Rho GTPase activity or actin polymerization not only prevents pore formation but also reduces effector translocation [22, 23]. These results indicate that the YopB/D-dependent activation of Rho in the host cell is required for effective translocation.

There is increasing data supporting the concept that in addition to recognizing pathogen-associated molecular patterns, the innate immune system can detect host cell processes induced by pathogens during infection [24, 25]. We have previously shown that during infection with a multi Yop mutant *Yersinia*, epithelial cells respond to the YopB/D translocon by mounting a proinflammatory response involving activation of mitogen-activated protein kinases (MAPKs), nuclear factor kappa B (NFκB) [25, 26]. This provided further evidence that the host cell recognizes the translocon. A YopB/D-dependent pro-inflammatory response was also demonstrated in infected macrophages lacking TLR receptors [21, 26–28].

YopD belongs to a family of structurally similar “minor translocators” [29]. The 306 amino acid YopD protein contains one central predicted transmembrane alpha helix region, a predicted C-terminal coiled-coil domain and a confirmed C-terminal amphipathic alpha helix [17, 30, 31].

It has been recently proposed that the YopB/D-mediated innate immune response elicited during *Yersinia* infection of macrophages is due to the recognition of an unidentified translocated PAMP and not by direct sensing of the translocon [32]. The study shows that a muti-Yop mutant induces TNF-alpha expression during infection of TLR4-deficient macrophages, while a translocation-deficient (YopDΔTM) mutant does not. However, the possibility that host cells specifically sense the YopB/D translocon could not be completely ruled out because the YopDΔTM mutant was also partly defective in pore formation, introducing smaller pores with delayed kinetics.

The aim of this work was to shed light into the multiple YopD functions and to study their individual role in virulence. Thus, we created a *yopD* mutant library to identify single amino acids required for translocation, pore formation or host cell signaling. We identified eight single amino acid substitutions that affected various YopD functions. Interestingly, three substitutions in a discrete C-terminal region significantly impaired pore formation, Yop translocation and host cell signaling without affecting YopD secretion, *yop* regulation, or YopD-YopB interaction. The role of these mutations *in vivo* was assessed in an well-established mouse model for *Yersinia pseudotuberculosis* [33]. We conclude that a previously unidentified domain located between the transmembrane and the putative coiled-coil domains at the C-terminal region of YopD is central for its function.

## Materials and Methods

### Bacterial strains

Wild type *Y*. *pseudotuberculosis* serogroup III strain and its derivatives carry a naturally occurring deletion in the virulence plasmid that inactivates the *yopT* gene (Table 1) [20]. Deletion of *yopD* in YP52 (*yopEHJD*), YP62 (*yopED*), YP63 (*yopD*) and IP63 (*yopD*) was performed by allelic exchange using suicide plasmid pSB890Δ*yopD* as follows. Recombinant PCR was used to construct Δ*yopD*, a 394bp DNA fragment containing the sequence corresponding to the 177 nucleotides upstream *yopD* fused in frame to the 217 bp immediately downstream *yopD*. The construct was created using the following primers: YopD1A (5′-TGAAGGATCCAGACATGGCAGCGTTAC-3′), YopD1B (5′-GGTTATTCCTCCTTAAACTTAAACAG-3′), YopD1C (5′-GGAGGAATAACC CCATTGATGACCTTGGT-3′), and YopD1D (5′-CGGTGGATCCCAGCTCAGGTCTTGA-3′). Underlined sequences correspond to BamHI restriction sites sequences. In bold is a tail sequence in primer YopD1C that overlaps with the first 12 nucleotides at the 5’ end of primer YopD1B. The resulting DNA fragment (Δ*yopD*) was cloned into BamHI-digested suicide plasmid pSB890. The resulting plasmid (pSB890Δ*yopD*) was introduced by electroporation into S17–1λpir, and transformants were selected on LB plates containing tetracycline. pSB890Δ*yopD* was transferred into YP27(Km^R^), YP6 (Km^R^), YP126 and IP2666, by conjugation. Transconjugants were selected on LB kanamycin tetracycline for YP27 and YP6, or *Yersinia* selective agar plates with tetracycline for YP126 and IP2666, Tet^R^ transconjugants were grown for several generations in the absence of tetracycline and then plated on LB agar plates lacking NaCl and supplemented with 5% sucrose to select against the *sacB* gene carried on pSB890. Sucrose-resistant colonies were tested for a Tet^S^ phenotype. The resulting mutants were verified by sequencing.

**Table 1:** Bacterial strains and plasmids.

Strain	Relevant features	Reference
YP126	Wild type Y. *pseudotuberculosis* YPIII, naturally YopT-deficient	[46]
IP2666	Wild type Y. *pseudotuberculosis* YPIII, naturally YopT-deficient	[33]
YP18	YP126*ΔyopB*	[46]
YP27	*yopH*::*cam*,*yopE*::*kan*,*ΔyopJ*	[47]
YP29	*yopH*::*cam*,*yopE*::*kan*,*ΔyopJ*,*ΔyopB*	[47]
YP52	*yopH*::*cam*,*yopE*::*kan*,*ΔyopJ ΔyopD*	This study
YP52/pYopD	YP52::*pYopD*	This study
YP6	*yopE*::*kan*	[48]
YP62	*yopE*::*kan*,*ΔyopD*	This study
YP62/pYopD	YP62::*pYopD*	This study
YP63	YP126*ΔyopD*	This study
YP63/pYopD	YP63::*pYopD*	This study
IP63	IP2666*ΔyopD*	This study
pMMB67HE	IPTG-inducible expression	[49]
pMMB67HEYopD	pMMB67HE::*yopD*	[26]
pSB890	Suicide plasmid Tet^R^, sacB	[46]
pSB890*ΔyopD*	pSB890::*ΔyopD* (177bp upstr *yopD* fused to 217bp downstr *yopD*)	This study
pSB890*yopD*_*AD*_	pSB890::*yopD* (−177bp *yopD* + 217bp)	This study

### Construction of a YopD mutant library

Error-prone PCR was used to create a library of single amino acid substitution in YopD. We used the GeneMorph II EZClone random mutagenesis kit from Stratagene that provides a mixture of two error prone DNA polymerases. *yopD* cloned into plasmid pMMB67HE (Amp^R^) was used as a template, and the following primers were used in the mutagenic PCR reaction: (pMMB67HE-Fw [5’ CGACATCATAACGGTTCTGGC 3’] and pMMB67HE-Rv [3’ GCGTTTCACTTCTGAGTTCGGC 3’]). These primers served to amplify a DNA fragment encompassing *yopD* and flanking pMMB67HE sequences (150bp upstream and 175bp downstream). As recommended to obtain 0–4.5 mutations/kb, the PCR reaction was performed using 100 ng of template DNA and 25 cycles. The amplified DNA was purified, digested with SacI and SphI, and cloned back into pMMB67HE to generate pYopD*. The pYopD* library was electroporated into competent YP62 (*yopE*::*kan*,*ΔyopD*) mutant and plated on LB supplemented with kanamycin (Kan) and carbenicillin (Car) to select for colonies containing the virulence plasmid and pYopD*, respectively. A total of 1144 Car^R^, Kan^R^ colonies were patched into 96-deep-well plate containing 0.4% agar LB supplemented with carbenicillin (Car) for maintenance at 4°C, and into LB glycerol to store at −20°C. Individual colonies of *yopED* and *yopED/*pYopD were included in each plate to serve as negative and positive control, respectively, in the screening tests.

### Native expression of I168T, G196R and A273T

*yopD* genes coding for I168T, G196R and A273T were expressed into YP52 and IP2666 by allelic replacement using pSB890-based plasmids, as described above. First, we created pSB890*yopD*_*1*AD_ by megaprimer PCR using pSB890*ΔyopD* as a template, and a 1.3kb PCR product comprising *yopD* plus its adjacent 177bp upstream and 217bp downstream regions (*yopD*_*1*AD_) as megaprimers. PCR product *yopD*_*1*AD_ was amplified from pYV using primers YopD1A and YopD1D (described above). Genes coding for YopDI168T, G196R and A273T were amplified from their pMMB67HE expression plasmid using primers YopDstart (ATGACAATAAATATCAAGAC) and YopDend (TCAGACAACACCAAAAGC). The resulting 921bp PCR products were purified and used as megaprimers to substitute *yopD* in pSB890*yopD*_*1*AD_ by PCR. The resulting plasmids were used to transform S17-λpir. Allelic replacement in YP63 and IP63 was performed as described above.

### Cell culture

HeLa cells (ATCC-CCL-2) were cultured in DMEM (Gibco BRL) supplemented with 10% heat-inactivated fetal calf serum (FCS; Gibco BRL) and 1 mM sodium pyruvate in a 5% CO2 humidified incubator at 37°C. For infection experiments performed to determine IL-8 production and LDH release, 1 × 10^5^ HeLa cells were seeded into wells of a 24-well tissue culture plate 24 h before assay. J774A.1 macrophage cell line origin (ATCC-TIB-67)

### Screening of the YopD* library

The ability of the YopD mutants to deliver Yops and to cause pore formation was tested simultaneously by assessing YopJ-mediated cell death in macrophages and osmotic lysis of HeLa cells, respectively. Overnight cultures were made by transferring *yopED*, *yopED/*pYopD and *yopED*/pYopD* colonies from a 0.4% agar LB 96-well plate to a 96-well plate containing 200 μl of LB broth supplemented with kanamycin and carbenicillin, except for the well containing *yopED*, in which only kanamycin was added. Incubation was carried out at 26°C with shaking in a moist chamber to prevent evaporation. The next day, bacteria were subcultured 1/20 in 96-well plate containing 200μl LB with 20 mM magnesium chloride and 20 mM *sodium oxalate* (low Ca^2+^ conditions) for 1 h at 26°C and 2 h at 37°C, with shaking. IPTG was added at the 37°C temperature shift to a final concentration of 50μM. To test for translocation, J774A.1 cells were cultured in 96 well plates at a density of 5x10^4^ cells/well and infected at an estimated MOI of 10 in the presence of IPTG. Gentamicin (100 μg/ml) was added 30 min after infection, and incubation proceeded overnight. LDH release from supernatants of infected cells was analyzed using CytoTox96 cytotoxicity assay kit (Promega). To test for pore formation, HeLa cells were cultured in 96 well plates at a density of 2x10^4^ cells/well and infected at a MOI of 100 in the presence of IPTG. Culture supernatants were collected 3 hours post infection and tested for LDH as described above.

### Yop secretion assay

Bacteria were cultured in 25 ml LB at low calcium conditions for 1 h at 26°C and 3 h at 37°C in the presence of 50μM IPTG. Proteins secreted in 1 ml of the culture supernatants were precipitated with 10% trichloroacetic acid (TCA), washed with ice-cold acetone, air-dried and suspended in Laemmli sample buffer. Bacterial pellet and secreted proteins were analyzed by Western blot using YopD Mab clone 248:19 [34] YopB mab clone 190.11 [35], and polyclonal antibodies against LcrV (provided by Matt Nilles, University of North Dakota) and YopH [36]. Anti-rabbit and anti-mouse IR680 or IR800 were used as secondary antibodies. Infrared signal was detected using the Odyssey imaging system (LI-COR Biosience).

### YopE-mediated cytotoxicity of HeLa cell

HeLa cells were infected at MOI of 100 for 1h with bacteria cultured under high calcium conditions, which repress the T3SS. Infected cells were examined by phase contrast using an inverted microscopy with a 40× objective. Images were recorded using a digital camera and were mounted using Adobe Photoshop CS5. Images are representative of three different experiments.

### LDH assay

HeLa cells in 24 well plates were infected with bacteria grown in LB broth under low Ca^2+^ condition at a multiplicity of infection of 100. The plates containing the infected cells were centrifuged for 5 min at 700 rpm and incubated at 37˚C with 5% CO_2_. Samples of culture media from wells containing infected cells were collected 3 hours post infection. Levels of LDH were assayed using the CytoTox 96 assay kit (Promega) as previously described. Results were normalized to *yopEHJD*/pYopD (100%). The mean and standard deviation values were obtained from four duplicate experiments.

### YopD co-immunoprecipitation

Bacterial supernatants were collected after 3 hours incubation at low calcium conditions with 50 μM IPTG, and incubated with 10 μg of YopD mab clone 248:19 with rocking for 1 hour at room temperature. The pellet of a 20μl Protein G-beads slurry (Dynabeads Invitrogen) was added to the immunocomplexes an incubated for 10 min while rocking. The beads were washed three times with PBS/0.01% Tween and resuspended in 2X Laemmli sample buffer, boiled for 5 min and resolved in SDS-PAGE and immunoblotting using anti-YopD 248:19 antibody and anti-YopB monoclonal antibody. Anti-rabbit and anti-mouse IR680 or IR800 were used as secondary antibodies. Infrared signal was detected and quantified using the Odyssey imaging system (LI-COR Biosience).

### Uptake of impermeant dyes

Hela cells cultured in 24-well plates with coverslips were infected for 3 h with bacteria grown under low calcium conditions. Uptake of ethidium homodimer-2 (EthD-2) by cells with compromised membranes was tested using the DEAD-LIVE kit (Invitrogen) following the protocol provided by the manufacturers. Cover slips were examined by using a fluorescence inverted microscopy with a 10× objective. Images were recorded using a digital camera and were mounted using Adobe Photoshop CS5.

### YopD-dependent activation of proinflammatory signaling

HeLa cells cultured in 6 well plates were infected at MOI 100 for 60 min, washed and lysed as previously described [26]. Immunoblotting was performed using polyclonal antibodies against phospho-ERK, p90RSK, IκBα (Cell Signaling) according to the procedures of the manufacturer. Monoclonal antibody 12G10 against α-tubulin from Developmental Studies Hybridoma Bank, was used as a loading control.

### Mouse infection

Eight-week-old female C57BL/6 mice (Jackson labs) were infected with *Y*. *pseudotuberculosis* wild type IP2666, IP2666YopDI168T, IP2666YopDG196R and IP2666YopDA273T. Mice were fasted for 18 h prior to infection. Bacterial inocula were prepared from overnight cultures grown in LB medium at 26°C, adjusted to 10^10^ cfu/ml and washed twice in PBS. In two independent experiments, 3 mice per group were infected orogastrically with 2x10^9^cfu (10 LD50) using a feeding needle and an inoculated volume of 0.2 ml. To record survival, infected mice were monitored three times a day, seven days a week over a 2-week period. Animals displaying serve signs of disease, characterized by hunched posture and immobility upon physical stimulation, were euthanized by CO_2_ inhalation to minimize suffering. These experiments were carried out in compliance with animal care and use regulations. The survival curve of each mutant was compared to that of the wild type infected mice and the statistical difference and P values were calculated using the Log-rank (Mantel Cox) test.

### Ethic Statement

The study was carried out in accordance with a protocol that adhered to the Guide for the Care and Use of Laboratory Animals of the National Institutes of Health (NIH) and was reviewed and approved (approval #206152) by the Institutional Animal Care and Use Committee at Stony Brook University, which operates under Assurance #A3011–01, approved by the NIH Office of Laboratory Animal Welfare.

## Results

### Construction and screening of a *yopD* mutant library

To better understand YopD function, we created a *yopD* library and screened for mutants that were secretion-competent but defective in translocation and/or pore formation. The library was generated using error prone PCR under conditions that created an average of one mutation per clone. The mutated PCR product was cloned into the expression vector pMMB67HE under the control of an IPTG-inducible promoter, resulting in pYopD*. The plasmid library was introduced into *E*. *coli* and subsequently moved to *Y*. *pseudotuberculosis* YPIII by conjugation. Because translocation of YopE or YopT during infection with wild type *Y*.*pseudotuberculosis* inhibits pore formation, and because YPIII naturally lacks YopT, we used a *yopED* mutant as a recipient of pYopD* (Table 1). This approach allowed for the simultaneous screening of YopJ translocation and pore formation. In addition, the kanamycin cassette inserted to disrupt the *yopE* gene in *yopED* mutant permitted the selection of colonies that maintained the virulence plasmid. The amount of YopD and other Yops secreted by *yopED*/pYopD at 500, 100 and 50 μM IPTG was compared to WT, *yopE* and *yopED* mutants (S1 Fig.). A high throughput genetic screening was implemented and 1144 colonies were tested. A concentration of 50 μM IPTG was used to induce YopD expression in cultures and during infections. Defects in Yop translocation and pore formation were assessed simultaneously in 96-well plates. Translocation of YopJ was indirectly measured by its ability to induce TLR4-mediated cell death in J774A.1 macrophages [37]. Percent of LDH release from infected cells was used to score cell death. Pore formation of infected HeLa cells leads to osmotic lysis, a phenotype that can also be measured by LDH release [20]. Because the readout for both assays is LDH release, we confirmed that under the infection conditions used in the macrophage cell death assay (low moi and short infection period before Gentamicin killing of extracellular bacteria, see Material and Methods) pore formation did not occur, i.e, infection with a pore forming strain that lacked YopJ (*yopEHJ*) did not cause LDH release from macrophages (data not shown).

**Fig 1:**
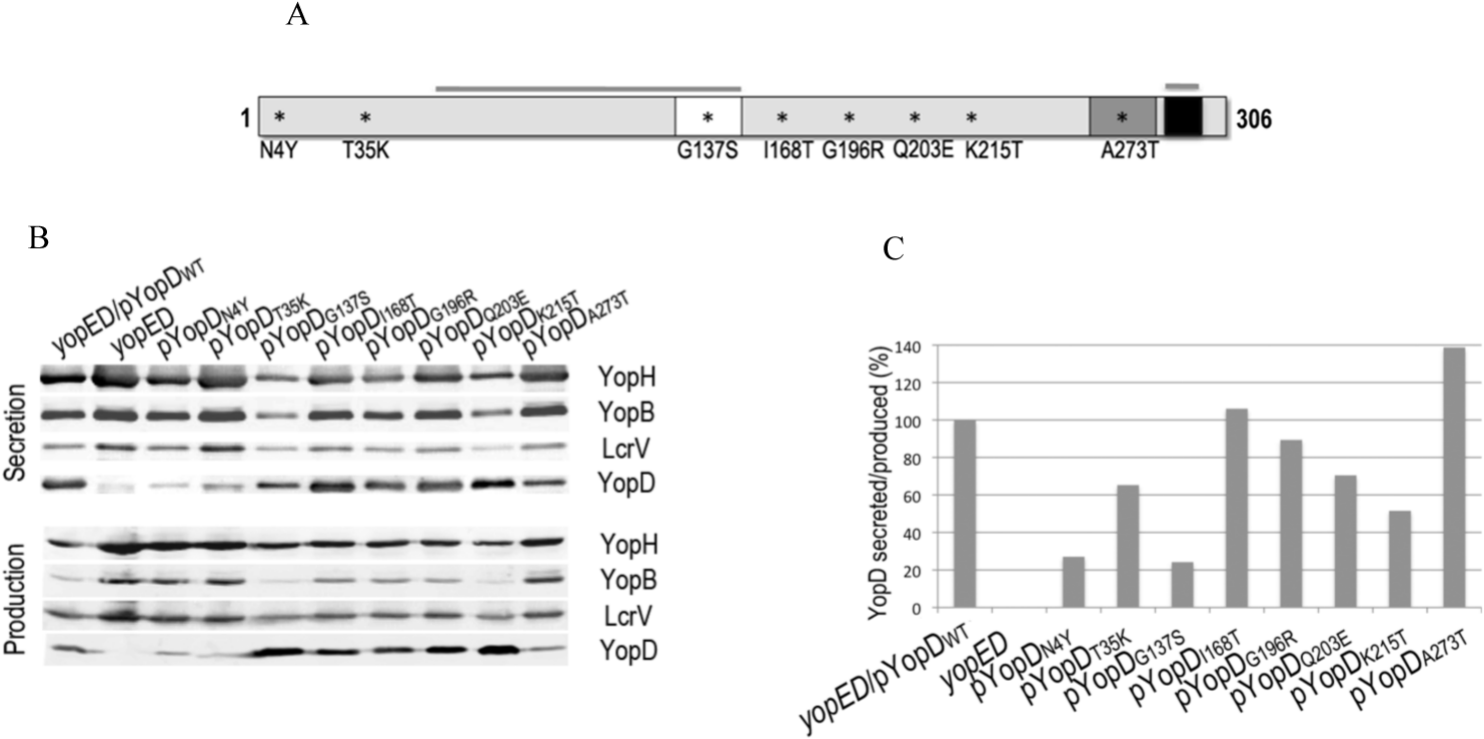
Secretion of the *yopD* mutants. **A**. Schematic representation of YopD protein indicating a predicted transmembrane domain (white), a putative coiled-coil region (dark grey) and an amphipathic α-helix domain (black). Overlined are the N-terminal and C-terminal chaperon-binding regions (53–149 and 278–292). Amino acid substitutions that interfere with pore formation, translocation or both are indicated by asterisks. **B**. Immunoblot showing Yop secretion (bacterial supernatant) and production (bacterial lysate) for each of the YopD mutants expressed in a *yopED* background and grown at 37°C at low calcium conditions. Monoclonal antibodies for the detection of YopD and YopB and polyclonal antibodies for YopH and LcrV are described in Material and Methods. Anti-rabbit and anti-mouse IR680 or IR800 were used as secondary antibodies. Infrared signal was detected using the Odyssey imaging system (LI-COR Biosience). Quantification of the YopD signal intensities was performed using Odyssey imaging system software. The ratio between the amount of YopD secreted and YopD produced was calculated for each mutant and normalized to wild type YopD.

Colonies with a defect in translocation, pore formation, or both, were subsequently tested for the ability of the mutants to secrete YopD. Those mutants with detectable amounts of secreted YopD by Western blot were subjected to DNA sequencing. We identified over 100 mutants that produced and secreted YopD but were defective in translocation, pore formation or both. Out of 100 mutants sequenced, 16 had a single missense mutation. The translocation, pore formation and YopD secretion phenotypes could be confirmed for 8 of these mutants. The location of the single amino acid substitution in each of those mutants is shown in Fig. 1A.

### Amino acid substitutions G137S and K215T interfere with calcium–dependent growth

*Yersinia* with *yopD* null mutations are defective in *yop* regulation and have a temperature-sensitive phenotype, growing poorly at 37°C irrespective of the calcium concentration. To determine whether any of the YopD amino acid substitutions affected CD growth, we cultured bacteria at low or high calcium concentration and measured OD_600_ every 30 minutes for 5 hours. Mutants with amino acid substitutions G137S and K215T exhibit a temperature sensitive phenotype similar to the *yopD* deletion mutant. The rest of the mutants displayed normal calcium-dependent growth (S2 Fig.). Henceforth we will refer to mutants G137S and K215T as Class I (Table 1).

### Substitutions N4Y, T35K and A273T affect YopD synthesis

To test the ability of each mutant to synthesize and secrete YopD, the protein profiles of whole bacteria cells and TCA-precipitated culture supernatants were determined after growth at low calcium conditions. Immunoblot assays using a monoclonal antibody against YopD showed that variable levels of YopD were present in bacterial pellets of the different mutants. Notably, mutants with YopD substitutions N4Y, T35K and A273T produced considerably less YopD than the WT, whereas substitutions G137S and K215T led to *yopD* overexpression (Fig. 1B).

### YopD secretion is decreased by N4Y and G137S residue exchange

To calculate the amount of YopD secreted by the different mutants, we measured the intensities of the YopD bands in the supernatant fractions of the immunoblot. Because of the variability in the amount of YopD produced, we calculated the ratio between secreted and produced YopD (Fig. 1B), and normalized it to that of the WT (Fig. 1C). While secretion of YopD N4Y and G137 were severely affected, the rest of the YopD variants were secreted at levels greater than or equal to 50% of the YopDWT.

### YopD mutants with normal calcium-dependent growth overproduced other Yops

We determined whether synthesis and secretion of other Yops were affected by the different YopD amino acid substitutions. As expected, *yopED*, which is temperature sensitive and defective in down-regulation of *yop* expression, produced increased amount of YopH, YopB, and LcrV, as determined by immunoblotting (Fig. 1B). The same pattern of Yop overproduction occurred in mutants with substitutions N4Y, T35K and A273T. As shown above, these mutants produced low amounts of YopD. Interestingly, these mutants display normal calcium-dependent growth. On the other hand, amino acid substitutions that affected normal calcium-dependent growth (G137S and K215T) resulted in a considerable downregulation of YopH, YopB and LcrVs synthesis (Fig. 1B). At high calcium conditions, G137S and K215T exhibited a wild type phenotype for Yop synthesis and secretion. This suggests that, at least under the conditions used in this assay, Yop regulation may be uncoupled from calcium-dependent growth in these mutants.

### T35K completely abrogates YopE-mediated rounding in Hela cells

**Fig 2:**
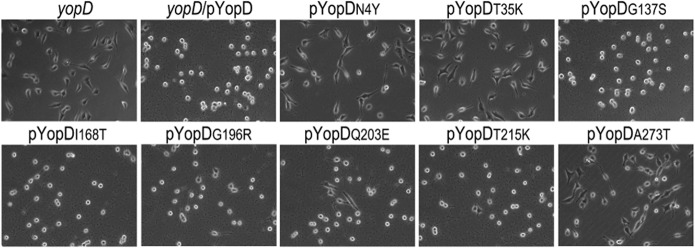
Effect of the different *yopD* mutations on translocation. **A.** *yopD* variants were expressed ectopically in a *yopD* single mutant strain and tested for YopE-mediated cell rounding of HeLa cells. Bacteria cultured under conditions that repress the T3SS were used to infect HeLa cells at MOI of 100 for 1h. YopE-mediated cytotoxicity was assessed by inverted microscopy using a 40× objective. Images were recorded using a digital camera. Images are representative of three different experiments.

Because the Rho GAP activity of YopE negatively controls translocation [22, 23], the lack of *yopE* causes the bacteria to translocate Yops at higher levels. To better assess the effect of the YopD substitutions in translocation, we transferred the pYopD* plasmids to a *yopD* background, and we assessed translocation by YopE-mediated rounding of infected HeLa cell. Because very low amounts of translocated YopE can cause disruption of the host cell cytoskeleton, we cultured bacteria under conditions that inhibit T3SS activation before bacterial-host cell contact. As expected, *yopD*/pYopD caused complete cell rounding one hour after infection, whereas no cytotoxicity was detected in cells infected with *yopD* mutant. YopD substitution T35K had a phenotype similar to *yopD*, whereas N4Y and A273T displayed some, but reduced levels of cytotoxicity (Fig. 2). The rest of the mutants caused cell rounding comparable to the wild type. A recognized caveat of the cytotoxicity assay is its poor ability to detect subtle defects in translocation. These results should be cautiously interpreted since we later found that the translocation phenotype of mutants I168T and G196R differed when the *yopD* mutations were introduced into the native *yopD* gene (see Fig. 6).

### Pore formation is compromised in mutants Class II and III

**Fig 3:**
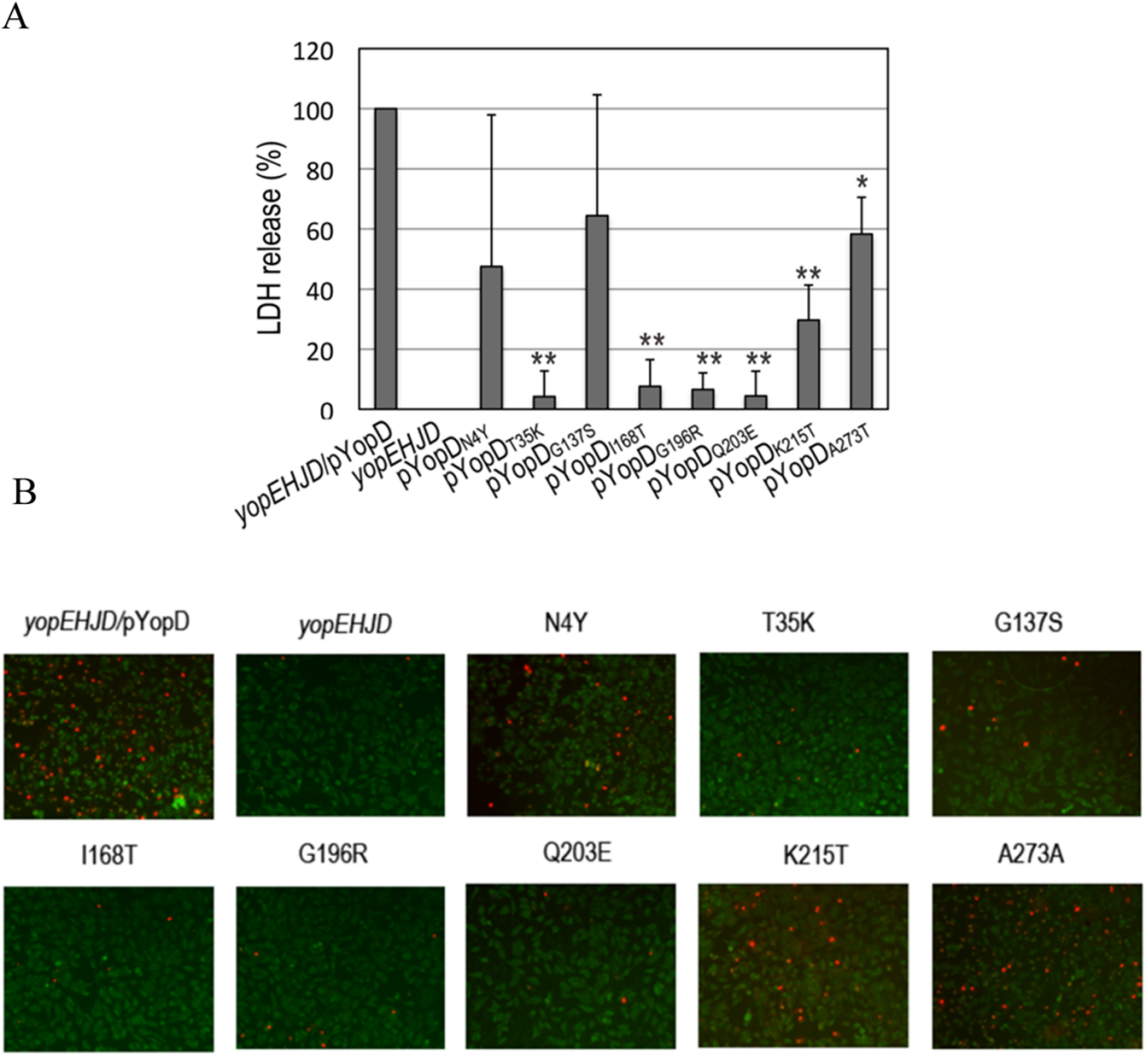
Effect of the different YopD mutations on pore formation. **A.** HeLa cells were left uninfected or infected with *yopEHJD*, *yopEHJD*/pYopD and *yopEHJD* expressing the different *yopD* mutants at MOI of 100. After 3 h infection, culture supernatants were removed and tested for LDH release. Background LDH released from uninfected cells was subtracted from infected wells. Results were normalized to *yopEHJD*/pYopD (100%). Error bars represent the standard deviation of the mean values obtained from four duplicate experiments. ** P<0.0001, * P<0.0005 determined by t-test. **B.** HeLa cells on coverslips were infected as described above for the LDH assay, and stained with DEAD-LIVE kit as described in Material and Methods. Cells with disrupted membranes exhibit a red nuclei staining.

The pore formation phenotype of the mutants selected in the screening was confirmed by infecting HeLa cells seeded on coverslips in 24-well plates at a MOI of 100. Because a *yopEHJ* mutant causes more pronounced pore formation than a single *yopE* mutant [20], we transferred pYopD* to a *yopEHJD* background. Three hours after infection, supernatants of uninfected and infected cells were tested for LDH release (Fig. 3A and S3 Fig.). All but N4Y and G137S were significantly defective in pore formation, with T35K, I168T, G196R and Q203E being the most severely affected. These results were confirmed by the uptake of DNA-binding impermeant dye ethidium homodimer-1 by infected HeLa cells (Fig. 3B). In this assay, only cells with compromised membranes take up the impermeant dye. Thus, infection with T35K, I168T, G196R and Q203E cause very low levels of pore formation as evidenced by only a few cells stained with red nuclei. As shown above, T35K was produced at much lower levels than YopDWT, whereas production and secretion of I168T, G196R and Q203E mutants were normal. We will therefore call pore formation-defective mutants T35K Class II, and I168T, G196R and Q203E Class III (Table 2).

### YopD mutants Class II and III fail to trigger a pro-inflammatory response

**Fig 4:**
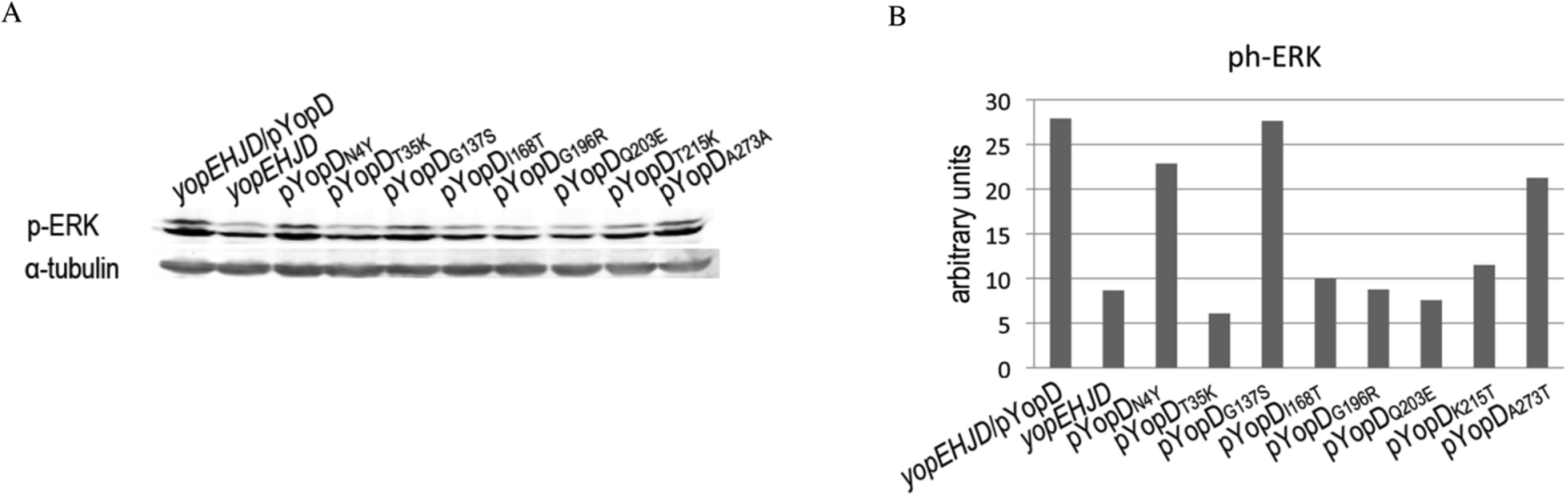
YopD-dependent activation of proinflammatory signaling. **A.** HeLa cells infected with *yopEHJD*, *yopEHJD*/pYopD and *yopEHJD* expressing the different YopD mutants at MOI of 100 for 1h. Cells were washed, lysed with sample buffer 1X, separated by SDS-PAGE and analyzed by immunoblotting with rabbit anti-phospho ERK. Monoclonal antibody against tubulin was used as a loading control. Quantification of the signal intensities was performed using Odyssey imaging system software. Values were normalized to tubulin.

**Fig 5:**
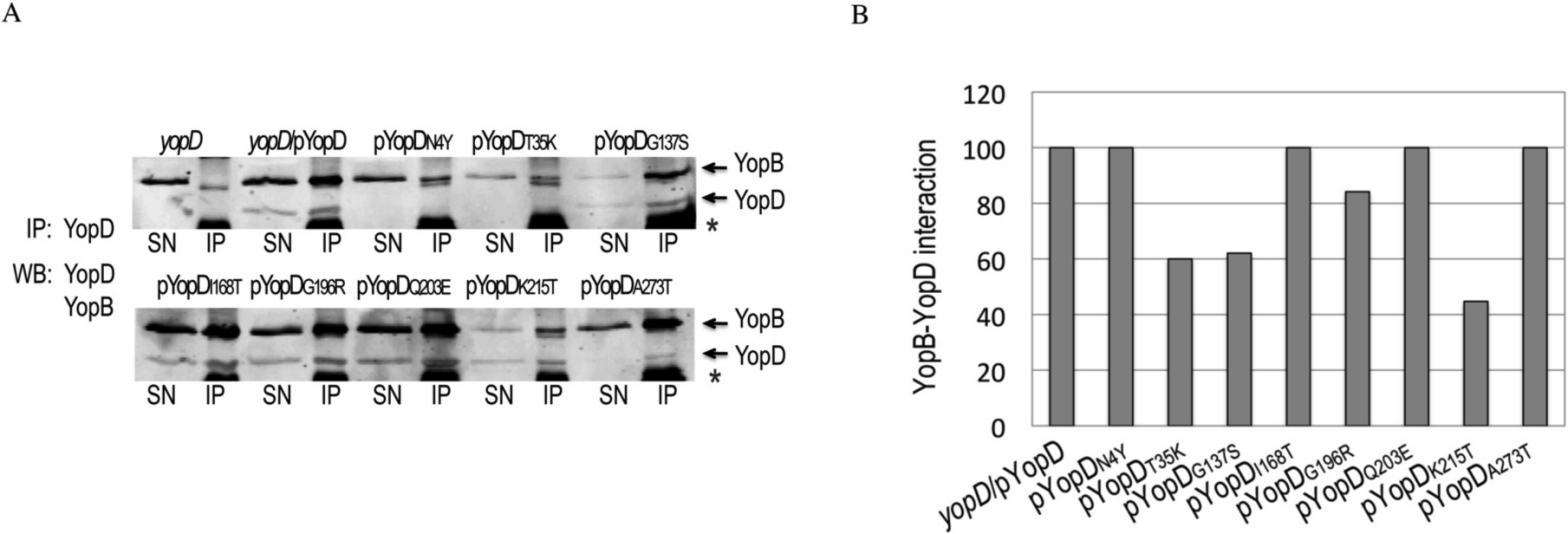
Interaction of the different YopD mutants with YopB. **A.** YopB-YopD complexes secreted in bacterial supernatants of *yopD*, *yopD*/pYopD and *yopD* expressing the different *yopD* mutants were precipitated by 1 h incubation with YopD mab (clone 248:19) followed by incubation with Dynabeads Protein G (Invitrogen). After 3 washes, beads were resuspended in 2X Laemmli sample buffer, and boiled. The eluted material (IP) and an aliquot of the bacterial supernatants (SN) were resolved in SDS-PAGE. Western blot was performed using anti-YopD and anti-YopB Mabs, and anti-mouse IR680 or IR800 secondary antibodies. Bands corresponding to YopB and YopD are indicated by arrows. Also present in the IP samples are a band originated from the beads (marked with an asterix), and a weak band of unknown origin that migrates right below YopB. **B.** Signal intensities of immunoprecipitated YopB and YopD were calculated using Odyssey imaging system software (LI-COR Biosience). YopB-YopD interaction for each mutant was calculated as the ratio between immunoprecipitated YopB and YopD. Results were normalized to *yopD*/pYopD.

Epithelial cells respond to infection with a *Yersinia* multi-yop mutant by triggering a series of pro-inflammatory signals [21, 26]. This response is dependent on the translocon and includes MAPKs and NFκB activation, and IL-8 production We have previously found that although a *yopEHJB* mutant does not trigger IL-8 production, infection with a *yopEHJD* mutant produces detectable levels of IL-8 [21, 26]. Therefore, at that time we concluded that YopD was dispensable for the proinflammatory response caused by the translocon. However, when we investigated the kinetics of MAPKs and NFκB activation we found that *yopEHJD* could not activate signal at early time points because, as shown in S4A Fig., levels of ph-ERK induced by *yopEHJD* and *yopEHJB* infection are comparable to those of uninfected cells. However, we could detect MAPKs and NFκB activation at 4-h infection (S4A Fig.). This delayed activation of proinflammatory signals could account for the modest IL-8 production found in our previous study. To determine what YopD residues are required for signal activation, we tested phosphorylation of the MAP kinase ERK in response to infection with *yopEHJD/*pYopD*. HeLa cells were left uninfected or infected for 1 h, and cell lysates were analyzed by immunoblotting using antibodies to ph-ERK (Fig. 4A and S5A Fig.). The ph-ERK signal intensities were quantified and the values were normalized to tubulin (Fig. 4B). Robust MAPK activation was observed when HeLa cells were infected with *yopEHJD*/pYopD, whereas much weaker MAPK phosphorylation was detected in cells infected with the *yopEHJD* mutant (Fig. 4). Class II (T35K) and Class III (I168T, G196R and Q203E) mutants were defective in MAPK signaling activation (Fig. 4). The results of the MAPK activation were confirmed by assessing the activation of p90RSK, a substrate of the MAPK/ERK (S5B and S5C Fig.). We also tested the effect of the different YopD amino acid substitutions on NFκB activation by measuring degradation of the NFκB inhibitor IκBα. IκBα degradation correlated with ERK activation (S5B Fig. and Fig. 5D). Intermediate levels of MAPK and NFκB activations were detected in cells infected with *yopEHJD*/pYopD N4Y, A273T and G137S. G137S belongs to TS Class I mutants. Because N4Y and A273T exhibit normal CD growth and have an intermediate pore formation and signaling phenotype, they are grouped as Class IV (Table 2).

### Interaction of YopD mutants with YopB

To determine whether the defect in translocation, pore formation or signaling was due to a defective interaction between the YopD variants and YopB we performed YopD co-immunoprecipitations on secreted Yops in culture supernatants. Fig. 5A shows that YopD monoclonal antibody effectively pulled down YopB from *yopED*/pYopD bacterial supernatant but not from that of *yopED*. Because the levels of YopB and YopD secretion vary among the different mutants, the degree of YopD-B interaction was expressed as the amount of pulled-down YopB relative to that of pulled-down YopD (Fig. 5B). With the exception of T35K, G137S and K215T (Class I and II), which exhibited 40–60% reduced YopB-YopD interaction, all the other YopD variants interacted with secreted YopB to high levels. Consistent with previous results in which secreted His-YopBDE complexes were purified from bacterial cultures using nickel affinity chromatography [38], YopD antibody also pulled down YopE, but not LcrV (not shown). Furthermore, a monoclonal antibody against LcrV also failed to pull down YopD from culture supernatants (not shown), suggesting that the YopBDE complex is not composed of aggregated Yops.

### Native expression of Class III mutants confirm multiple defects in translocon function

To determine whether the phenotypes of the *yopD* mutants could be confirmed when expressed from the native *yopD* gene, we selected three *yopD* mutants with normal calcium dependent growth; two with strong pore formation and signaling defects representing Class III (I168T, G196R) and one with a more intermediate phenotype from Class IV (A273T). Thus, the three *yopD* mutations were moved to its native location in *yopEHJD* (YP52), as described in Material and Methods, and the resulting strains were tested in parallel for translocation, pore formation and MAPK activation.

**Fig 6:**
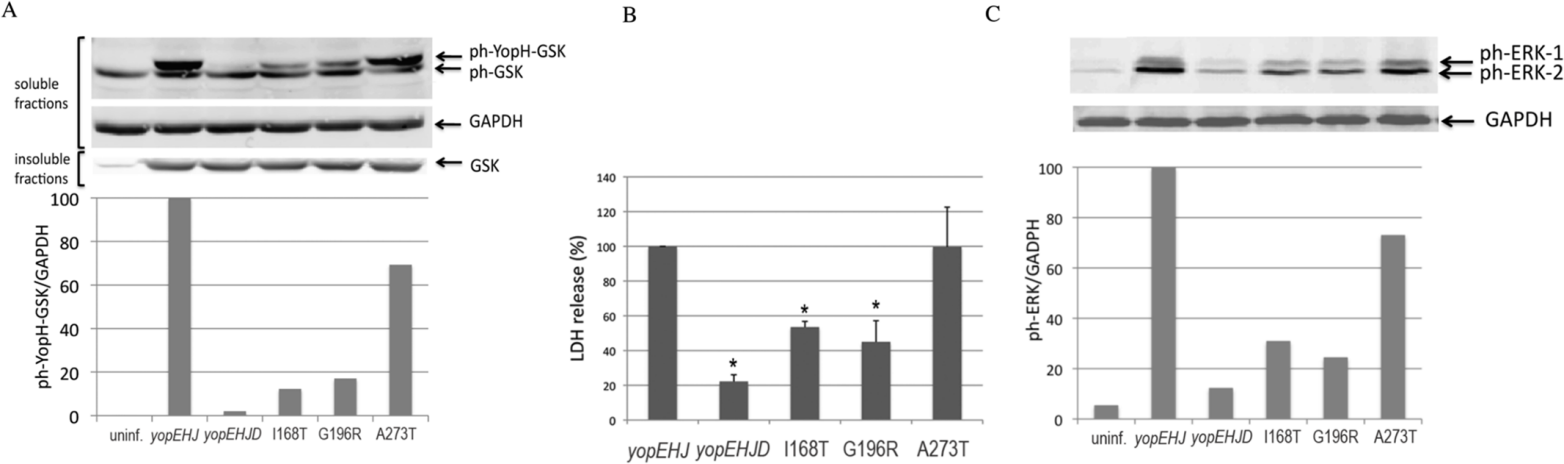
Phenotype of the *Y*. *pseudotuberculosis* expressing *yopD* mutations in its native location. HeLa cells were infected with *yopEHJ*, *yopEHJD*, and *yopEHJD* expressing *yopD*I168T, G196R and A273T in their native location. **A.** Effector translocation was determined by infecting HeLa cells at a MOI of 100 for 2h. Cells were washed, lysed with 1% Triton X100, and soluble and insoluble fractions were separated by SDS-PAGE and analyzed by immunoblotting with rabbit anti-phospho-*GSK*-3β (Ser9). Monoclonal antibody against GAPDH and total *GSK*-3β (Ser9) antibody were used as a loading control for the soluble and insoluble fractions, respectively. Quantification of the signal intensities was performed using Odyssey imaging system software. Results are expressed as the ratio between total ph-ERK signal and GAPDH. **B.** Pore formation was determined as described in Fig. 4 by analyzing the amount of LDH released from culture supernatants of infected cells. Results were normalized to *yopEHJ* (100%). Error bars represent the standard deviation of the mean values obtained from three duplicate experiments. * P<0.005 determined by t-test. **C.** YopD-dependent activation of proinflammatory signaling was performed as described in Fig. 5. MAPK activation was analyzed in cell lysates by immunoblotting using rabbit anti-phospho ERK. A monoclonal antibody against GAPDH was used as a loading control and quantification of the signal intensities was performed using Odyssey imaging system software. Results are expressed as the ratio between total ph-ERK signal and GAPDH.

To quantitatively test for effector translocation in these mutants, we used the glycogen synthase kinase GSK peptide tag-based reporter system [39]. When the tagged effector protein is translocated into the eukaryotic cell, the GSK peptide is phosphorylated by host cell kinases. The phosphorylated fusion protein can then be detected in the cell lysate by immunoblot using an anti ph-GSK antibody. Thus, *yopEHJD* (YP52) strains expressing the *yopD* mutations in their native location were transformed with the arabinose-inducible expression plasmid pBAD*yopH-gsk* by electroporation. Arabinose (0.2%) was added to the bacterial cultures and was kept during infection of HeLa cells to induce the expression of YopH-gsk fusion protein. Two hours after infection, cells were washed with PBS, and lysed with 1%Triton X-100. Soluble and insoluble fractions of uninfected and infected cells were analyzed by immunoblotting using antibodies to phospho-*GSK*-3β (Ser9). Eukaryotic glyceraldehyde 3-phosphate dehydrogenase (GAPDH) signal was used as a loading control of the soluble fraction, and total GSK as a control for the insoluble fraction. A strong band of phosphorylated YopH-GSK was detected in cells infected with *yopEHJ* and *yopEHJ* expressing *yopD* A273T, whereas much less translocation occurred in cells infected with *yopEHJ* expressing Class III *yopD* mutations I168T and G196R (Fig. 6A). These results did not correlate with the translocation phenotypes assessed by HeLa cell rounding when the mutations were expressed ectopically (Fig. 2).

In parallel, the same strains were tested for pore formation activity by LDH release in HeLa cells 3 hours post infection. Although the phenotypes were less dramatic than those observed with the ectopic expression of these YopD mutants, the defect in pore formation could be confirmed for Class III mutants I168T and G196R (Fig. 6B).

MAPK activation was also assessed as described above, by infecting HeLa cells for 1 h and analyzing cell lysates by immunoblotting using antibodies to ph-ERK. In accordance to what was observed when the *yopD* mutations were expressed ectopically, infection with *yopEHJ*YopDA273T elicited a strong ERK phosphorylation, while a poorer MAPK activation, was detected upon infection with mutants Class III I168T and G196R (Fig. 6C).

Based on these results we postulate that YopD amino acid I168 and G196, and most likely also Q203, constitute a new domain in YopD important for translocation, pore formation, and host signaling.

### In vivo studies

**Fig 7:**
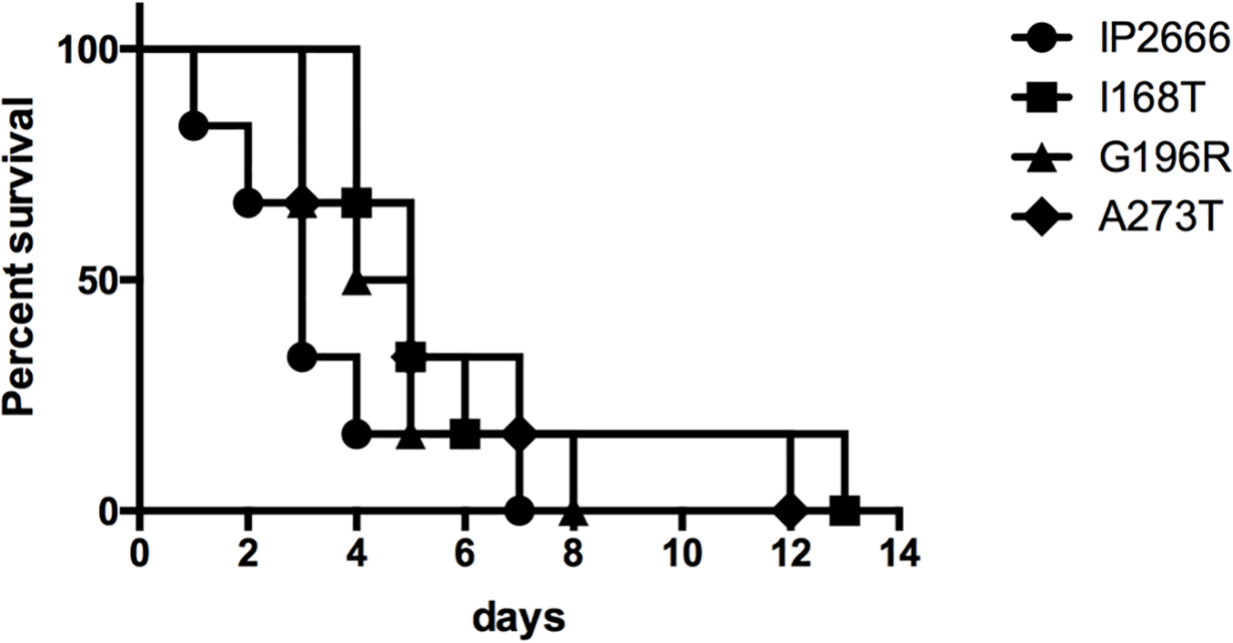
Residues I168, G196 and A273 are not required for virulence of *Y*. *pseudotuberculosis* IP2666 in a mouse model. Eight-week old C57BL/6J mice were infected orogastrically with 2×10^9^ CFU of wild type *Y*. *pseudotuberculosis* IP2666 and its derivative expressing *yopD* I168T, G196R and A273T. Mouse survival was monitored for 14 days. Results shown are pooled from two independent experiments using 3 mice per group in each experiment. Statistical analysis using log rank test showed no significant differences between the survival curves of each of the mutants and that of the wild type.

To determine if substitution I168T or G196R would impact virulence, we infected mice orogastrically with wild type *Y*. *pseudotuberculosis* IP2666, IP2666 natively expressing I168T, G196R and, as a control, A273T. Secretion of YopD and other Yops was not affected by the endogenous expression of the *yopD* variants (S6 Fig.). After infection, survival was followed up for 2 weeks. The survival of mice infected with *IP2666* expressing any of the *yopD* mutations was not significantly different from that of the mice infected with wild type IP2666 (I168T: χ^2^ 2.51, P = 0.1131; G196R: χ^2^ 1.59, P = 0.2077; A273T: χ^2^ 2.3, P = 0.1297, Fig. 7). These results indicate that although substitutions I168T and G196R affect translocation, pore formation and epithelial cell recognition *in vitro*, these amino acids changes do not decrease virulence under the conditions used in this mouse model.

## Discussion

YopD plays a multifaceted role in *Yersinia* virulence. Inside the bacterial cell YopD is in complex with its chaperon and, in cooperation with YscM1/LcrQ, prevents *yop* transcription during non-permissive condition for Yop secretion. Once secreted, YopD interacts with itself and YopB to form a multimeric ring that can insert in lipid membranes [6]. Insertion of this translocation channel in the membrane of the host cell has several consequences, i.e. it mediates delivery of effectors inside the host cell, it triggers Rho GTPase activation, it leads to cell lysis if the effector Yops do not inhibit Rho GTPases activation [20] [18], and it is directly or indirectly sensed by the innate immune system to mount a pro-inflammatory response [20, 21, 25, 32]. We propose that epithelial cells’ activation of Rho GTPase by the translocon might be detected as a “pattern of pathogenesis”.

Frequently, translocation-deficient *yopD* mutants also show a defect in Yop synthesis regulation, complicating the study of such mutants. Here we have used a genetic screen to isolate randomly generated YopD variants defective in YopD function. This comprehensive mutational analysis allowed the identification of four different types of mutants (Table 2).

**Table 2:** Phenotypes of the four different classes of YopD mutants.

Class	Mutation	Growth type	YopD production	YopD secretion	Pore formation	Signal
**I**	G137S	TS	high	low	interm	interm
	K215T	TS	high	normal	interm	interm
**II**	T35K	CD	low	normal	low	low
**III**	I168T	CD	normal	normal	low	low
	G196R	CD	normal	normal	low	low
	Q203E	CD	normal	normal	low	low
**IV**	N4Y	CD	low	low	interm	interm
	A273T	CD	low	low	interm	interm

Summary of YopD mutants and their phenotypes compared to wild type YopD. Growth type was determined at 37°C with and without calcium, “TS” refers to temperature sensitive, and “CD” to 37°C calcium-dependent growth; YopD production and secretion were determined in a bacterial culture at low calcium conditions in the pellet and supernatants, respectively; pore formation was tested in HeLa cells by LDH release and uptake of impermeant dyes; signal activation of MAPK and NFκB was assessed in HeLa cells. “interm” refers to intermediate phenotype.

Six *yopD* mutants (Class II-IV) have normal low calcium response but are defective in translocon function. Class II and III mutants have the strongest phenotype. Importantly, mutants in Class III have amino acid substitutions in a discrete region located between the transmembrane and the putative C-terminal coiled-coil domain (residues 168 to 203). This region has been found to be partly unfolded in several YopD-homologues. Besides allowing YopD’s secretion through the needle, this disordered structure is thought to be required for its interaction with host cell partners [29]. Significantly, residues I168, G196 and Q203 are conserved among YopD homologues in *Pseudomonas*, *Aeromonas* and *Photorhabdus* species (S7 Fig.). The amino acid substitutions in this region range from radical in Q203E (uncharged to acidic), and G196R (uncharged to basic) to semi-conservative in I168T (hydrophobic to polar) Independently, the library screening identified other mutants within the 168–203 region that were excluded from the study because they exhibited more than one missense mutation. Among them was a mutant that resulted in a hydrophobic to polar amino acid substitution at position 168 (I168N), suggesting that the hydrophobic residue at position 168 may play an important role in translocon function. Three others such mutants included YopD variants with substitutions in amino acids 181, 183 and 185 (not shown). Although the consequences of these residues changes would have to be tested independent from the other mutations, it is possible that those amino acids are also part of this previously unrecognized distinct region within the C-terminus of YopD that is required for translocation, pore formation, and activation of the MAPK and NFκB pathways in HeLa cells.

We have previously shown that Yop effectors prevent membrane damage by counteracting a signal triggered by the insertion of the translocon [20]. This signal involves Rho GTPase activation and leads to localized actin polymerization. We postulate that counteracting this signal is essential for the host cell membrane resealing process to prevent cell lysis. We envision that when translocation pores are not promptly sealed, impermeant dyes can enter the cell. At this point, the membrane permeabilization process is reversible. If, for some reason, the membrane cannot be repaired, osmotic lysis occurs and the cytoplasmic content is released. Consistent with this model is our unpublished observation that infection of HeLa cells with a single *yopK* mutant, which presumably inserts larger pores [40], results in strong membrane blebbing and high uptake of impermeant dyes. However, because *yopK* mutant can translocate Yops the process can be reversed, cell lysis does not occur, and LDH is not released. The transient inhibition of membrane resealing might be necessary to facilitate delivery of effectors. Supporting this model is our previous data showing that inhibition of Rho GTPases or actin polymerization decreases effector translocation, and conversely loss of YopE-mediated GAP activity leads to hypertranslocation [22, 23]. We reason that substitutions I168T, G196S and Q203E lead to defects in Rho GTPase signaling that impact translocation and pore formation. Because these mutants also failed to trigger MAPK, our model predicts that it is this very signal that is recognized as a pattern of pathogenesis by host cell to trigger an inflammatory response. Different scenarios can explain the defect in signaling. One possibility is that this C-terminal domain is by itself involved in Rho-GTPase activation. Interestingly, that region has been shown to interact with the cytoplasm of the host cell in the enteropathogenic *E*. *coli* YopD homologue EspB, suggesting that these residues could directly or indirectly interact with host cell signal proteins [41]. Another possibility is that Rho activation requires correct membrane insertion of a properly assembled YopB/D channel. Although results from the immuno-precipitation studies suggest that I168T, G196S and Q203E do not affect interaction between secreted YopB and YopD, it is possible that different interactions are required to form a signal-competent structure. It should be noted that the role of this domain in *Yersinia* infection is uncertain, as results from orogastrically-infected mice indicate that substitutions I168T and G196R, do not significantly affect virulence. It was surprising to find that mutants with a marked defect in translocation were not attenuated. On the other hand, it still possible that subtle differences in virulence could not be demonstrated with the conditions used in our *in vivo* studies.

Substitution T35K has dramatic effects on the translocation and pore formation functions of YopD. The lower level of T35K secretion is not likely to account for those defects as even lower levels of secreted YopD were identified in N4Y and yet this mutant is fully capable of causing pore formation. A serious defect in the interaction between and T35K and YopB can also be ruled out by the results obtained from the immuno-precipitation assay. Thus, although at lower levels than wild type YopD, T35K was able to pull down YopB from culture supernatants. Alternatively, T35K substitution could potentially be disrupting an N-terminal coiled-coil (CC) structure predicted with by Costa *et al* using the COILS server [42]. In that low probability CC region, threonine 35 would correspond to the *d* residue from a heptad repeat hxxhcxc, of hydrophobic (h) and charged (c) amino-acid residues. When we used the same program to test whether a threonine to lysine substitution at position 35 would disrupt such a structure, the probability of forming a CC domain did not change. This suggests that the YopD defects caused by the T35K substitution might not be attributed to the disruption of a putative N-terminal CC domain. More studies would need to be done to determine the cause of the T35K substitution’s dramatic phenotype.

Class I mutants exhibit a temperature sensitive growth. Mutants defective in the low calcium response produce Yops constitutively and have a temperature sensitive growth at 37°C irrespective of the calcium concentrations [9, 17]. Previous comprehensive sequential in-frame deletion mutagenesis studies of YopD have identified two main regions required for regulatory control: one spanning residues 53 to 149 and the other 278 to 292 [17]. This regions overlaps with domains required for interaction of YopD with its chaperone LcrH/SycD. Glycine 137 is located within the amino-terminal LcrH/SycD binding site. Lysine 215, however, is an area of the protein not previously associated with regulation. As the LcrH/SycD is required for stabilization of its substrates and both mutants are produced at high levels, it is unlikely that these mutants have a defect in their interaction with their chaperone. It is possible that G137S and K215T substitutions interfere with normal calcium dependent growth by preventing YopD interaction with another component of the T3SS required for normal calcium-dependent growth. These YopD partners might include YscV, which has been identified as a suppressor of the temperature-sensitive mutation in YopD [12].

Substitutions G137S and K215T also lead to hyperproduction of YopD and, interestingly, these mutants produced markedly reduced amounts of YopB. Because YopB and YopD share the LcrH/SycD chaperone, this phenomenon might be the result of a competition for a common chaperon. Although a single chaperone molecule could conceivably bind to both translocators simultaneously, data from the crystal structure of a YopD peptide bound to LcrH/SycD suggests that this is not likely the case [43]. Interestingly, inconsistent to what it would be expected if mutants with substitutions G137S and K215T had a defect in *yop* regulation, expression of LcrV and YopH was not increased in those mutants. These results suggest that in these mutants calcium dependent growth can be uncoupled from *yop* regulation.

A predicted transmembrane domain has been identified between residues 128 to 149 of YopD [17, 44]. Although G137 is located within the transmembrane domain, the Glycine to Serine substitution did not significantly affect translocation or pore formation. Accordingly, results obtained using the prediction of transmembrane regions and orientation from the EMBnet server, indicate that the G137S substitution is not likely to affect the transmembrane domain (not shown). The moderate defect in pore formation and translocation caused by G137S and K215T YopD substitutions might simply be attributed to the lower amount of YopB and LcrV produced and secreted by those mutants.

When expressed ectopically, N4Y and A273T showed a translocation defect, whereas their pore formation was not considerably reduced. We were not expecting to identify mutants defective in either translocation or pore formation, as we envision the two processes to be connected. Previous studies have identified mutants defective exclusively in translocation [17]. These studies analyzed pore formation by infection of red blood cells with bacteria that lacked YopK, which supposedly insert much larger pores [40]. Here, we have tested pore formation in HeLa cells using uptake of Ethidium homodimer and LDH release. YopD mutants previously reported to cause lysis in red blood cells, such as YopDΔ53–68 and Δ128–149 [17], test negative in our two pore formation assays (data not shown). So, clearly, these two assays measure different cellular events. Notably, when A273T was expressed in it native location translocation levels were restored to close to 70% of that of the WT. This suggests that slight differences in the amount of protein produced when *yopD* is expressed ectopically versus in its native location may affect the proper pore stoichiometry.

Asparagine at position 4 lies within a previously identified N-terminal YopD secretion signal [45]. We have found that this residue exchange not only impacts secretion, as it would be expected, but most noticeably, it reduces YopD production. A possible role of the 5′ end of *yopD* in translation control has been suggested previously [45]. Although decreased levels of YopD production were also detected in the A273T substitution, secretion was not as greatly affected as N4Y, which is expected, as the C-terminus does not seem to be involved in secretion.

Although more work needs to be done to fully understand their role in T3S, the mutants identified in this genetic screening provide a useful tool to further explore the different functions of YopD.

## Supporting Information

S1 Fig IPTG titration. Wild type (YP126), *yopE* (YP6), *yopED* (YP62) and *yopED*/pYopD bacterial cultures were grown at 37°C at low calcium conditions with different concentrations of IPTG added. Equivalent amounts of cultures supernatants containing secreted Yops were resolved by SDS-PAGE and the gel was stained with Coomassie Blue. Positions of molecular weight standards (kDa) are indicated on the left and bands corresponding to the different Yops are indicated on the right.
(TIF)

S2 Fig Growth curves at low and high calcium conditions. Yersinia *yopED*, *yopED*/pYopD and *yopED* expressing the different *yopD* variants were cultured in LB broth at low calcium (LC) and high calcium (HC) at 37°C for 5 hours. Absorbance at OD_600_ was measured every 30 minutes and growth curves were constructed for each mutant.
(TIF)

S3 Fig Effect of the different YopD mutations on pore formation. Pore formation was determined as described in Fig. 3 by analyzing the amount of LDH released from culture supernatants of uninfected and infected cells. Results from a typical experiment before normalization and subtraction of the LDH content present in uninfected wells are shown. LDH release is expressed in arbitrary units.
(TIF)

S4 Fig Kinetics of MAPK activation. A. HeLa cells were left uninfected or infected with *yopEHJ*, *yopEHJB* and *yopEHJD* for 1hour. Cell lysates were separated by SDS-PAGE and analyzed by immunoblotting with rabbit anti-phospho ERK, and anti-rabbit IR680. Monoclonal antibody against tubulin was used as a loading control (not shown). Quantification of the signal intensities was performed using Odyssey imaging system software. Values were normalized to tubulin. B. Kinetics of MAPK activation was tested at 1, 2 and 4 hours post infection. Cell lysates were analyzed by immunoblotting with rabbit anti-phospho ERK, and anti-rabbit IR680, and anti-IκBα and anti-rabbit IR680.
(TIF)

S5 Fig YopD-dependent activation of proinflammatory signaling. **A.** HeLa cells were left uninfected or infected with *yopEHJD*, and *yopEHJD*/pYopD at MOI of 100 for 1h. Cells were washed, lysed with sample buffer 1X, separated by SDS-PAGE and analyzed by immunoblotting with rabbit anti-phospho ERK. Monoclonal antibody against tubulin was used as a loading control (not shown). **B.** HeLa cells infected with *yopEHJD*, *yopEHJD*/pYopD and *yopEHJD* expressing the different *yopD* mutants at the same conditions as described above. Immunoblotting was performed with rabbit anti-phospho p90RSK, anti-IκBα, and anti-tubulin, independently. C **and D.** Quantification of the signal intensities was performed using Odyssey imaging system software. Values were normalized to tubulin.
(TIF)

S6 Fig Yop secretion of strains used for animal studies. Comassie Blue stained SDS-PAGE gel showing Yop secretion for IP2666 (WT) *yopD* (IP63) and IP63 endogenously expressing YopDI168T, G196R and A273Y and grown at 37°C at low calcium conditions.
(TIF)

S7 Fig Sequence alignment. A 55 amino acid region of YopD encompassing residues 168–203, was aligned with the corresponding protein regions of three other YopD homologues: *Pseudomonas aeruginosa*, *Aeromonas* spp. and *Photorhabdus* spp. Identical residues are shown in bold.
(TIF)

## References

[1] Buttner D. Protein export according to schedule: architecture, assembly, and regulation of type III secretion systems from plant- and animal-pathogenic bacteria. Microbiol Mol Biol Rev. 2012 Jun;76(2):262–310. 10.1128/MMBR.05017-11 22688814 PMC3372255

[2] Viboud GI, Bliska JB. Yersinia outer proteins: role in modulation of host cell signaling responses and pathogenesis. Annu Rev Microbiol. 2005;59:69–89. 15847602 10.1146/annurev.micro.59.030804.121320

[3] Sory MP, Cornelis GR. Translocation of a hybrid YopE-adenylate cyclase from Yersinia enterocolitica into HeLa cells. Mol Microbiol. 1994 Nov;14(3):583–94. 7885236 10.1111/j.1365-2958.1994.tb02191.x

[4] Rosqvist R, Magnusson KE, Wolf-Watz H. Target cell contact triggers expression and polarized transfer of Yersinia YopE cytotoxin into mammalian cells. EMBO J. 1994 Feb 15;13(4):964–72. 8112310 10.1002/j.1460-2075.1994.tb06341.x PMC394898

[5] Persson C, Nordfelth R, Holmstrom A, Hakansson S, Rosqvist R, Wolf-Watz H. Cell-surface-bound Yersinia translocate the protein tyrosine phosphatase YopH by a polarized mechanism into the target cell. Mol Microbiol. 1995 Oct;18(1):135–50. 8596454 10.1111/j.1365-2958.1995.mmi_18010135.x

[6] Montagner C, Arquint C, Cornelis GR. Translocators YopB and YopD from Yersinia enterocolitica form a multimeric integral membrane complex in eukaryotic cell membranes. J Bacteriol. 2011 Dec;193(24):6923–8. 10.1128/JB.05555-11 22001511 PMC3232856

[7] Mueller CA, Broz P, Cornelis GR. The type III secretion system tip complex and translocon. Mol Microbiol. 2008 Jun;68(5):1085–95. 10.1111/j.1365-2958.2008.06237.x 18430138

[8] Thanassi DG, Bliska JB, Christie PJ. Surface organelles assembled by secretion systems of Gram-negative bacteria: diversity in structure and function. FEMS Microbiol Rev. 2012 Nov;36(6):1046–82. 10.1111/j.1574-6976.2012.00342.x 22545799 PMC3421059

[9] Williams AW, Straley SC. YopD of Yersinia pestis plays a role in negative regulation of the low-calcium response in addition to its role in translocation of Yops. J Bacteriol. 1998 Jan;180(2):350–8. 9440524 10.1128/jb.180.2.350-358.1998 PMC106890

[10] Francis MS, Lloyd SA, Wolf-Watz H. The type III secretion chaperone LcrH co-operates with YopD to establish a negative, regulatory loop for control of Yop synthesis in Yersinia pseudotuberculosis. Mol Microbiol. 2001 Nov;42(4):1075–93. 11737648 10.1046/j.1365-2958.2001.02702.x

[11] Wattiau P, Bernier B, Deslee P, Michiels T, Cornelis GR. Individual chaperones required for Yop secretion by Yersinia. Proc Natl Acad Sci U S A. 1994 Oct 25;91(22):10493–7. 7937981 10.1073/pnas.91.22.10493 PMC45047

[12] Fowler JM, Wulff CR, Straley SC, Brubaker RR. Growth of calcium-blind mutants of Yersinia pestis at 37 degrees C in permissive Ca2+-deficient environments. Microbiology. 2009 Aug;155(Pt 8):2509–21. 10.1099/mic.0.028852-0 19443541 PMC2888125

[13] Anderson DM, Ramamurthi KS, Tam C, Schneewind O. YopD and LcrH regulate expression of Yersinia enterocolitica YopQ by a posttranscriptional mechanism and bind to yopQ RNA. J Bacteriol. 2002 Mar;184(5):1287–95. 11844757 10.1128/JB.184.5.1287-1295.2002 PMC134855

[14] Kopaskie KS, Ligtenberg KG, Schneewind O. Translational regulation of Yersinia enterocolitica mRNA encoding a type III secretion substrate. J Biol Chem. 2013 Dec 6;288(49):35478–88. 10.1074/jbc.M113.504811 24158443 PMC3853294

[15] Chen Y, Anderson DM. Expression hierarchy in the Yersinia type III secretion system established through YopD recognition of RNA. Mol Microbiol. 2011 May;80(4):966–80. 10.1111/j.1365-2958.2011.07623.x 21481017 PMC4128491

[16] Francis MS, Aili M, Wiklund ML, Wolf-Watz H. A study of the YopD-lcrH interaction from Yersinia pseudotuberculosis reveals a role for hydrophobic residues within the amphipathic domain of YopD. Mol Microbiol. 2000 Oct;38(1):85–102. 11029692 10.1046/j.1365-2958.2000.02112.x

[17] Olsson J, Edqvist PJ, Broms JE, Forsberg A, Wolf-Watz H, Francis MS. The YopD translocator of Yersinia pseudotuberculosis is a multifunctional protein comprised of discrete domains. J Bacteriol. 2004 Jul;186(13):4110–23. 15205412 10.1128/JB.186.13.4110-4123.2004 PMC421591

[18] Hakansson S, Schesser K, Persson C, Galyov EE, Rosqvist R, Homble F, et al The YopB protein of Yersinia pseudotuberculosis is essential for the translocation of Yop effector proteins across the target cell plasma membrane and displays a contact-dependent membrane disrupting activity. EMBO J. 1996 Nov 1;15(21):5812–23. 8918459 PMC452329

[19] Neyt C, Cornelis GR. Insertion of a Yop translocation pore into the macrophage plasma membrane by Yersinia enterocolitica: requirement for translocators YopB and YopD, but not LcrG. Mol Microbiol. 1999 Sep;33(5):971–81. 10476031 10.1046/j.1365-2958.1999.01537.x

[20] Viboud GI, Bliska JB. A bacterial type III secretion system inhibits actin polymerization to prevent pore formation in host cell membranes. EMBO J. 2001 Oct 1;20(19):5373–82. 11574469 10.1093/emboj/20.19.5373 PMC125656

[21] Viboud GI, Mejia E, Bliska JB. Comparison of YopE and YopT activities in counteracting host signalling responses to Yersinia pseudotuberculosis infection. Cell Microbiol. 2006 Sep;8(9):1504–15. 16922868 10.1111/j.1462-5822.2006.00729.x

[22] Mejia E, Bliska JB, Viboud GI. Yersinia controls type III effector delivery into host cells by modulating Rho activity. PLoS Pathog. 2008 Jan;4(1):e3 10.1371/journal.ppat.0040003 18193942 PMC2186360

[23] Aili M, Isaksson EL, Carlsson SE, Wolf-Watz H, Rosqvist R, Francis MS. Regulation of Yersinia Yop-effector delivery by translocated YopE. Int J Med Microbiol. 2008 Apr;298(3–4):183–92. 10.1016/j.ijmm.2008.04.005 17597003

[24] Vance RE, Isberg RR, Portnoy DA. Patterns of pathogenesis: discrimination of pathogenic and nonpathogenic microbes by the innate immune system. Cell Host Microbe. 2009 Jul 23;6(1):10–21. 10.1016/j.chom.2009.06.007 19616762 PMC2777727

[25] Bliska JB, Wang X, Viboud GI, Brodsky IE. Modulation of innate immune responses by Yersinia type III secretion system translocators and effectors. Cell Microbiol. 2013 Oct;15(10):1622–31. 10.1111/cmi.12164 23834311 PMC3788085

[26] Viboud GI, So SS, Ryndak MB, Bliska JB. Proinflammatory signalling stimulated by the type III translocation factor YopB is counteracted by multiple effectors in epithelial cells infected with Yersinia pseudotuberculosis. Mol Microbiol. 2003 Mar;47(5):1305–15. 12603736 10.1046/j.1365-2958.2003.03350.x

[27] Auerbuch V, Golenbock DT, Isberg RR. Innate immune recognition of Yersinia pseudotuberculosis type III secretion. PLoS Pathog. 2009 Dec;5(12):e1000686 10.1371/journal.ppat.1000686 19997504 PMC2779593

[28] Brodsky IE, Palm NW, Sadanand S, Ryndak MB, Sutterwala FS, Flavell RA, et al A Yersinia effector protein promotes virulence by preventing inflammasome recognition of the type III secretion system. Cell Host Microbe. 2010 May 20;7(5):376–87. 10.1016/j.chom.2010.04.009 20478539 PMC2883865

[29] Mattei PJ, Faudry E, Job V, Izore T, Attree I, Dessen A. Membrane targeting and pore formation by the type III secretion system translocon. FEBS J. 2011 Feb;278(3):414–26. 10.1111/j.1742-4658.2010.07974.x 21182592

[30] Costa TR, Edqvist PJ, Broms JE, Ahlund MK, Forsberg A, Francis MS. YopD self-assembly and binding to LcrV facilitate type III secretion activity by Yersinia pseudotuberculosis. J Biol Chem. 2010 Aug 13;285(33):25269–84. 10.1074/jbc.M110.144311 20525687 PMC2919090

[31] Wang T, Diaz-Rosales P, Costa MM, Campbell S, Snow M, Collet B, et al Functional characterization of a nonmammalian IL-21: rainbow trout Oncorhynchus mykiss IL-21 upregulates the expression of the Th cell signature cytokines IFN-gamma, IL-10, and IL-22. J Immunol. 2011 Jan 15;186(2):708–21. 10.4049/jimmunol.1001203 21160047

[32] Kwuan L, Adams W, Auerbuch V. Impact of host membrane pore formation by the Yersinia pseudotuberculosis type III secretion system on the macrophage innate immune response. Infect Immun. 2013 Mar;81(3):905–14. 10.1128/IAI.01014-12 23297383 PMC3584892

[33] Simonet M, Falkow S. Invasin expression in Yersinia pseudotuberculosis. Infect Immun. 1992 Oct;60(10):4414–7. 1398952 10.1128/iai.60.10.4414-4417.1992 PMC257481

[34] Noel BL, Lilo S, Capurso D, Hill J, Bliska JB. Yersinia pestis can bypass protective antibodies to LcrV and activation with gamma interferon to survive and induce apoptosis in murine macrophages. Clin Vaccine Immunol. 2009 Oct;16(10):1457–66. 10.1128/CVI.00172-09 19710295 PMC2756853

[35] Chung LK, Philip NH, Schmidt VA, Koller A, Strowig T, Flavell RA, et al IQGAP1 is important for activation of caspase-1 in macrophages and is targeted by Yersinia pestis type III effector YopM. MBio. 2014;5(4):e01402–14. 10.1128/mBio.01402-14 24987096 PMC4161239

[36] Montagna LG, Ivanov MI, Bliska JB. Identification of residues in the N-terminal domain of the Yersinia tyrosine phosphatase that are critical for substrate recognition. J Biol Chem. 2001 Feb 16;276(7):5005–11. 11069923 10.1074/jbc.M009045200

[37] Ryndak MB, Chung H, London E, Bliska JB. Role of predicted transmembrane domains for type III translocation, pore formation, and signaling by the Yersinia pseudotuberculosis YopB protein. Infect Immun. 2005 Apr;73(4):2433–43. 15784589 10.1128/IAI.73.4.2433-2443.2005 PMC1087397

[38] Ivanov MI, Noel BL, Rampersaud R, Mena P, Benach JL, Bliska JB. Vaccination of mice with a Yop translocon complex elicits antibodies that are protective against infection with F1- Yersinia pestis. Infect Immun. 2008 Nov;76(11):5181–90. 10.1128/IAI.00189-08 18765742 PMC2573372

[39] Garcia JT, Ferracci F, Jackson MW, Joseph SS, Pattis I, Plano LR, et al Measurement of effector protein injection by type III and type IV secretion systems by using a 13-residue phosphorylatable glycogen synthase kinase tag. Infect Immun. 2006 Oct;74(10):5645–57. 16988240 10.1128/IAI.00690-06 PMC1594927

[40] Holmstrom A, Petterson J, Rosqvist R, Hakansson S, Tafazoli F, Fallman M, et al YopK of Yersinia pseudotuberculosis controls translocation of Yop effectors across the eukaryotic cell membrane. Mol Microbiol. 1997 Apr;24(1):73–91. 9140967 10.1046/j.1365-2958.1997.3211681.x

[41] Luo W, Donnenberg MS. Interactions and predicted host membrane topology of the enteropathogenic Escherichia coli translocator protein EspB. J Bacteriol. 2011 Jun;193(12):2972–80. 10.1128/JB.00153-11 21498649 PMC3133209

[42] Costa TR, Amer AA, Fallman M, Fahlgren A, Francis MS. Coiled-coils in the YopD translocator family: A predicted structure unique to the YopD N-terminus contributes to full virulence of Yersinia pseudotuberculosis. Infect Genet Evol. 2012 Dec;12(8):1729–42. 10.1016/j.meegid.2012.07.016 22910185

[43] Schreiner M, Niemann HH. Crystal structure of the Yersinia enterocolitica type III secretion chaperone SycD in complex with a peptide of the minor translocator YopD. BMC Struct Biol. 2012;12:13 10.1186/1472-6807-12-13 22708907 PMC3443056

[44] Hakansson S, Bergman T, Vanooteghem JC, Cornelis G, Wolf-Watz H. YopB and YopD constitute a novel class of Yersinia Yop proteins. Infect Immun. 1993 Jan;61(1):71–80. 8418066 10.1128/iai.61.1.71-80.1993 PMC302689

[45] Amer AA, Ahlund MK, Broms JE, Forsberg A, Francis MS. Impact of the N-terminal secretor domain on YopD translocator function in Yersinia pseudotuberculosis type III secretion. J Bacteriol. 2011 Dec;193(23):6683–700. 10.1128/JB.00210-11 21965570 PMC3232875

[46] Palmer LE, Hobbie S, Galan JE, Bliska JB. YopJ of Yersinia pseudotuberculosis is required for the inhibition of macrophage TNF-alpha production and downregulation of the MAP kinases p38 and JNK. Mol Microbiol. 1998 Mar;27(5):953–65. 9535085 10.1046/j.1365-2958.1998.00740.x

[47] Palmer LE, Pancetti AR, Greenberg S, Bliska JB. YopJ of Yersinia spp. is sufficient to cause downregulation of multiple mitogen-activated protein kinases in eukaryotic cells. Infect Immun. 1999 Feb;67(2):708–16. 9916081 10.1128/iai.67.2.708-716.1999 PMC96377

[48] Bliska JB, Galan JE, Falkow S. Signal transduction in the mammalian cell during bacterial attachment and entry. Cell. 1993 Jun 4;73(5):903–20. 8500180 10.1016/0092-8674(93)90270-z

[49] Furste JP, Pansegrau W, Frank R, Blocker H, Scholz P, Bagdasarian M, et al Molecular cloning of the plasmid RP4 primase region in a multi-host-range tacP expression vector. Gene. 1986;48(1):119–31. 3549457 10.1016/0378-1119(86)90358-6

