# Correction: Random Mutagenesis Identifies a C-Terminal Region of YopD Important for *Yersinia* Type III Secretion Function

**DOI:** 10.1371/journal.pone.0130112

**Published:** 2015-06-12

**Authors:** The PLOS ONE Staff

## Notice of Republication

This article was republished on May 15, 2015, to correct a Unicode character in the seventh sentence of the Abstract: “NFκB” should be “NFκB.” The publisher apologizes for the error. Please download this article again to view the correct version.
