# Supplementary figures and images for: Random Mutagenesis Identifies a C-Terminal Region of YopD Important for Yersinia Type III Secretion Function
Source: PLoS One. 2015 Mar 25;10(3):e0120471. doi: 10.1371/journal.pone.0120471 (PMC4433470; doi:10.1371/journal.pone.0120471)

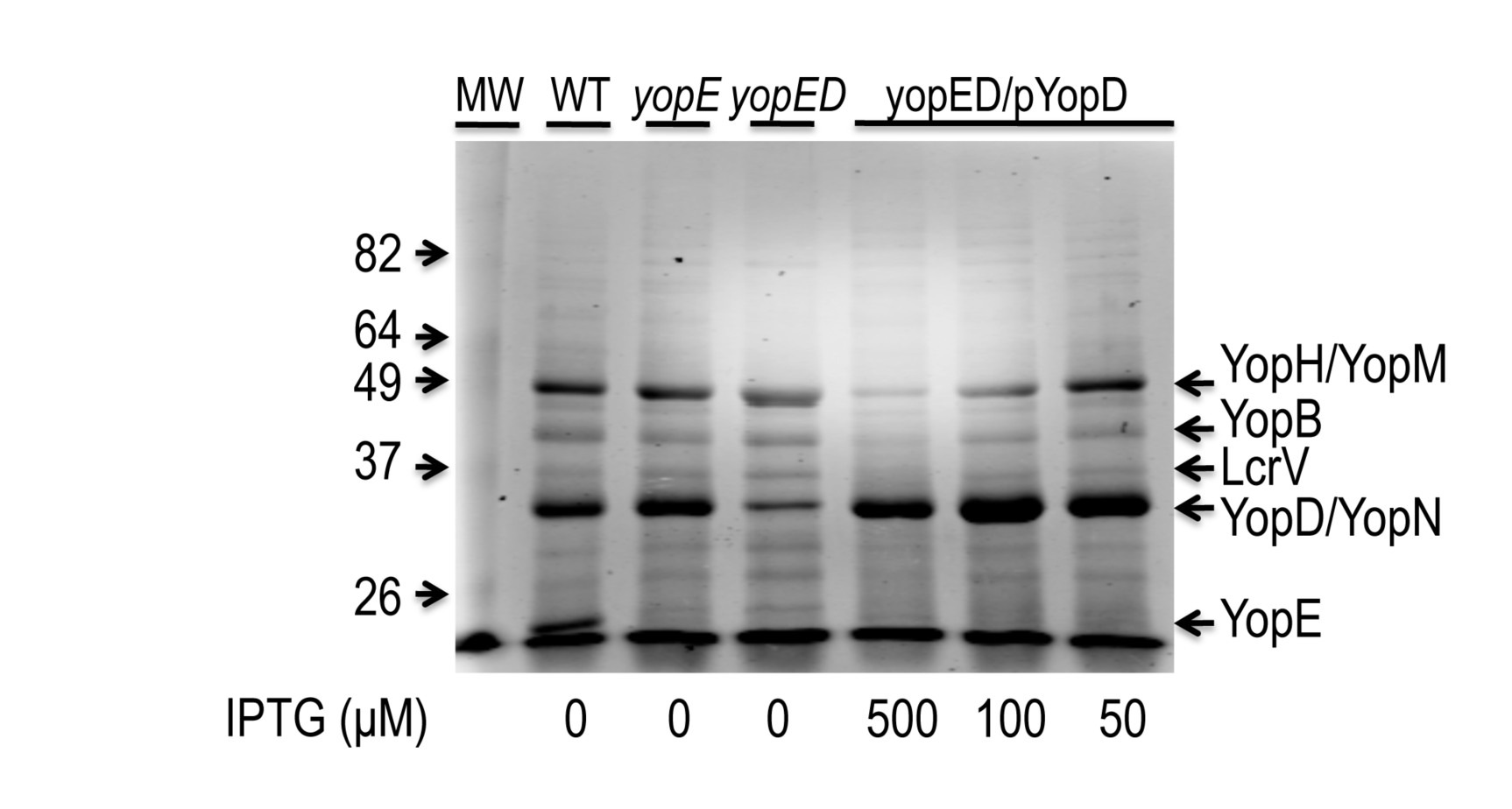

Supplement: S1 Fig — Wild type (YP126), yopE (YP6), yopED (YP62) and yopED/pYopD bacterial cultures were grown at 37°C at low calcium conditions with different concentrations of IPTG added. Equivalent amounts of cultures supernatants containing secreted Yops were resolved by SDS-PAGE and the gel was stained with Coomassie Blue. Positions of molecular weight standards (kDa) are indicated on the left and bands corresponding to the different Yops are indicated on the right. (TIF) [file pone.0120471.s001.tif]

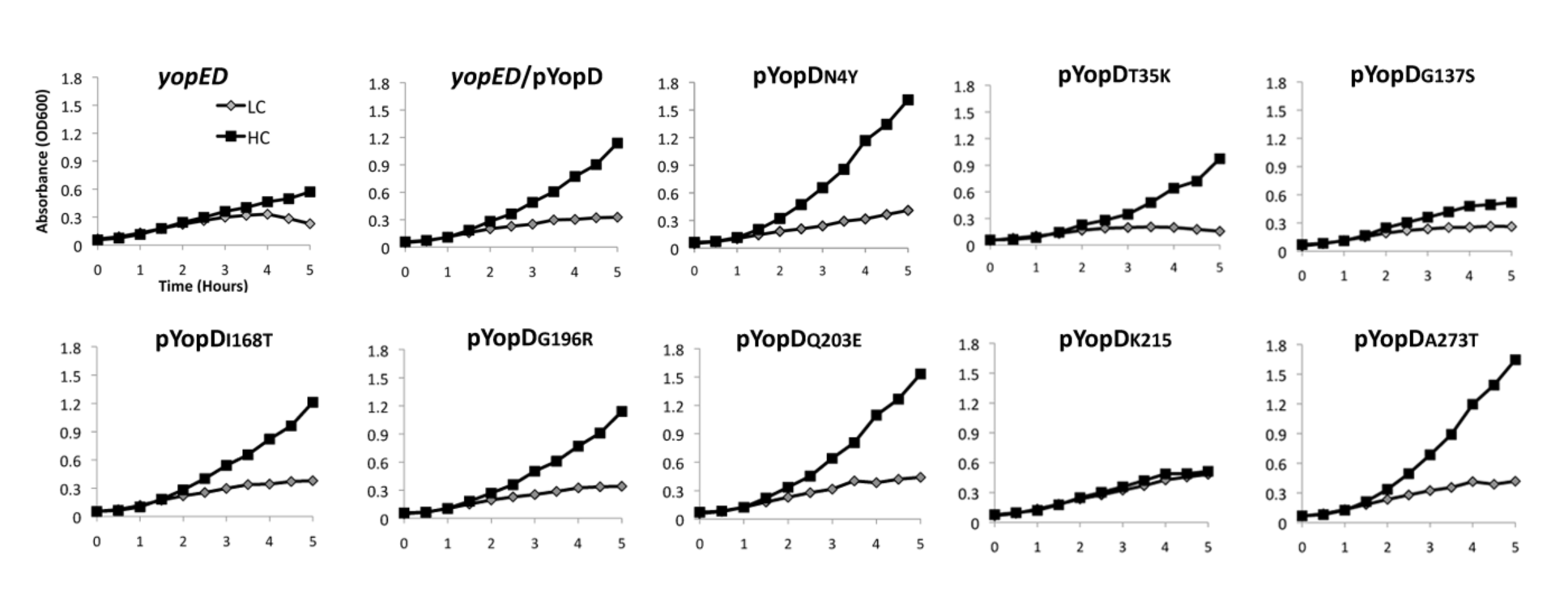

Supplement: S2 Fig — Yersinia yopED, yopED/pYopD and yopED expressing the different yopD variants were cultured in LB broth at low calcium (LC) and high calcium (HC) at 37°C for 5 hours. Absorbance at OD600 was measured every 30 minutes and growth curves were constructed for each mutant. (TIF) [file pone.0120471.s002.tif]

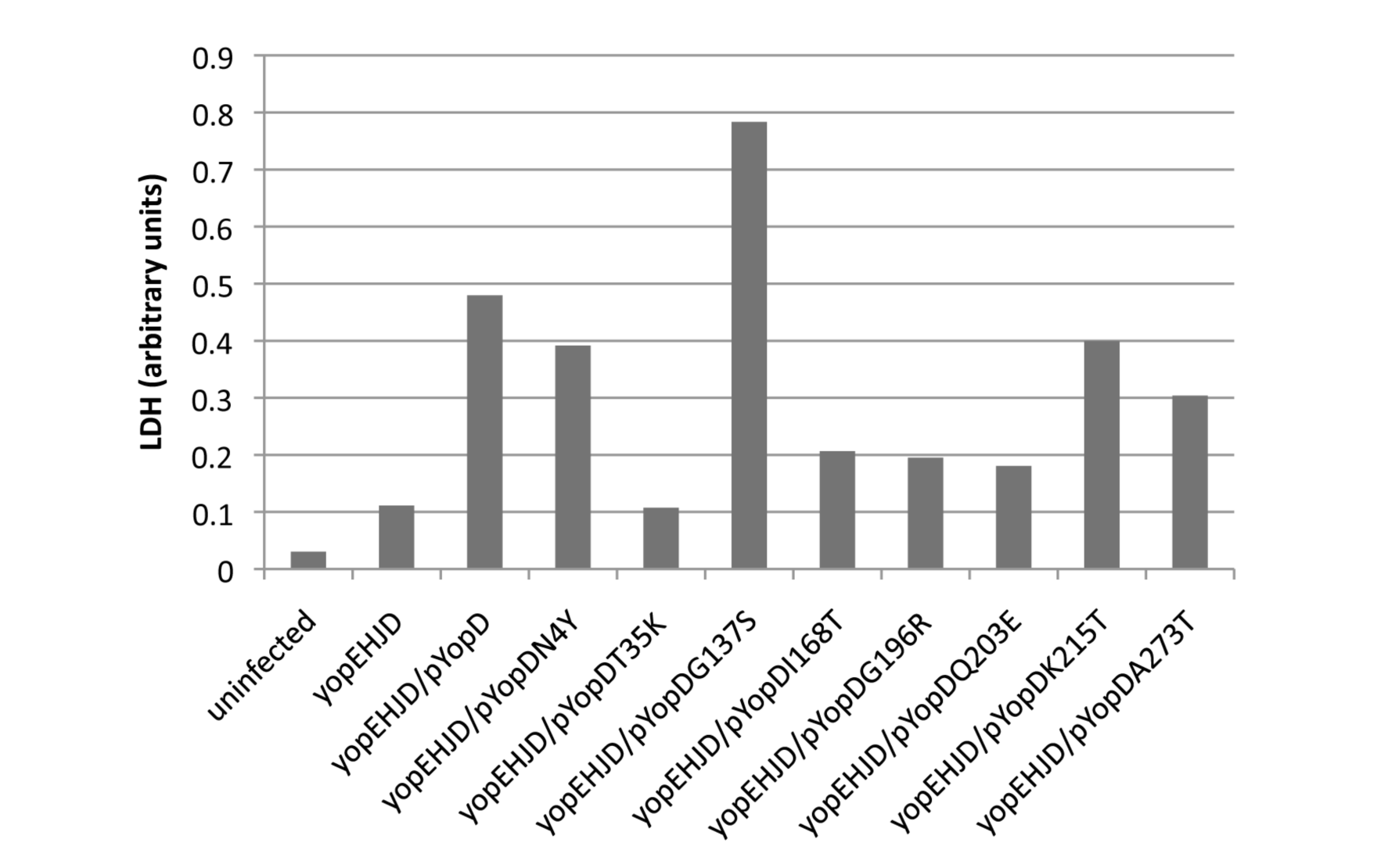

Supplement: S3 Fig — Pore formation was determined as described in Fig. 3 by analyzing the amount of LDH released from culture supernatants of uninfected and infected cells. Results from a typical experiment before normalization and subtraction of the LDH content present in uninfected wells are shown. LDH release is expressed in arbitrary units. (TIF) [file pone.0120471.s003.tif]

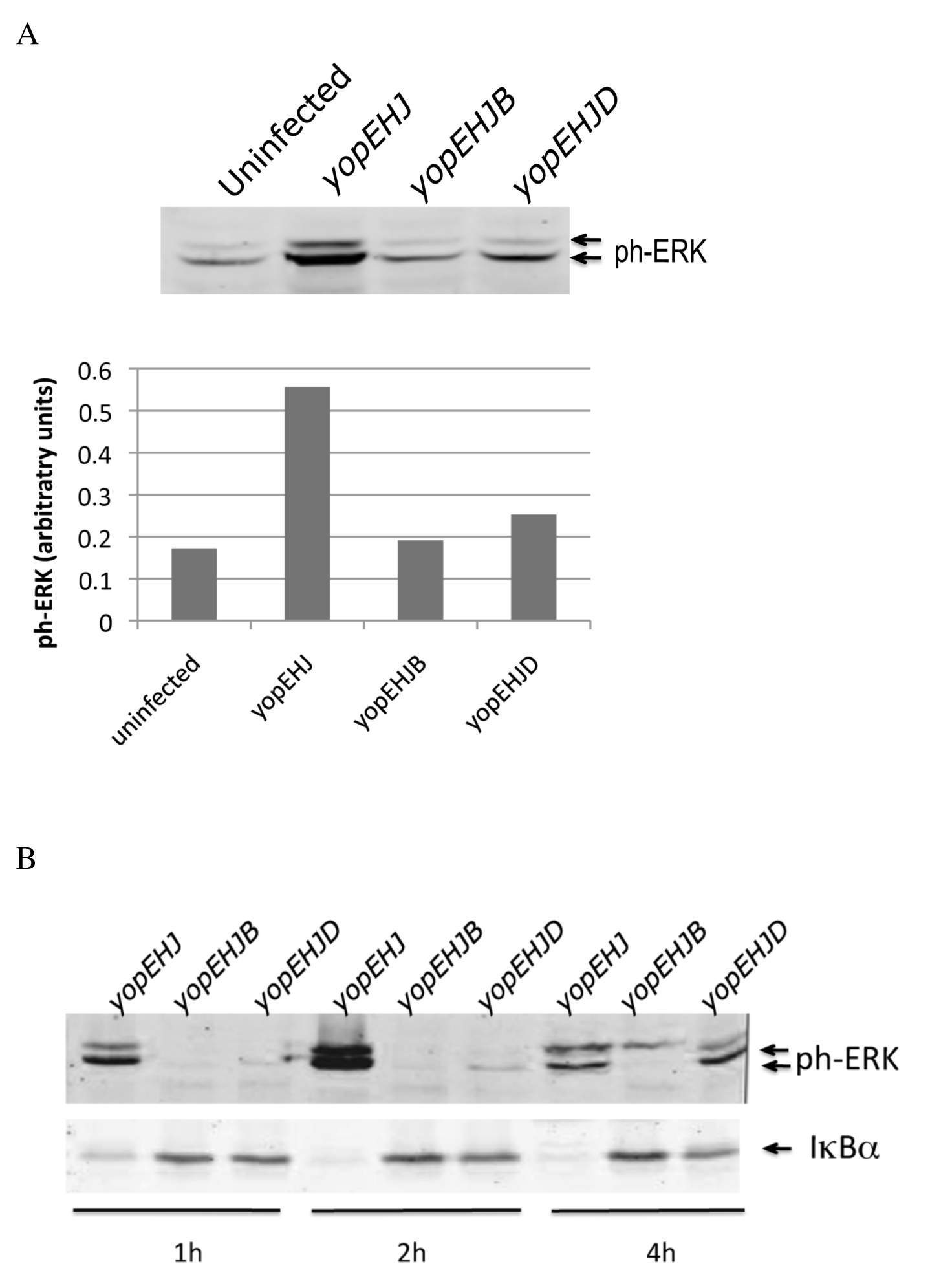

Supplement: S4 Fig — A. HeLa cells were left uninfected or infected with yopEHJ, yopEHJB and yopEHJD for 1hour. Cell lysates were separated by SDS-PAGE and analyzed by immunoblotting with rabbit anti-phospho ERK, and anti-rabbit IR680. Monoclonal antibody against tubulin was used as a loading control (not shown). Quantification of the signal intensities was performed using Odyssey imaging system software. Values were normalized to tubulin. B. Kinetics of MAPK activation was tested at 1, 2 and 4 hours post infection. Cell lysates were analyzed by immunoblotting with rabbit anti-phospho ERK, and anti-rabbit IR680, and anti-IκBα and anti-rabbit IR680. (TIF) [file pone.0120471.s004.tif]

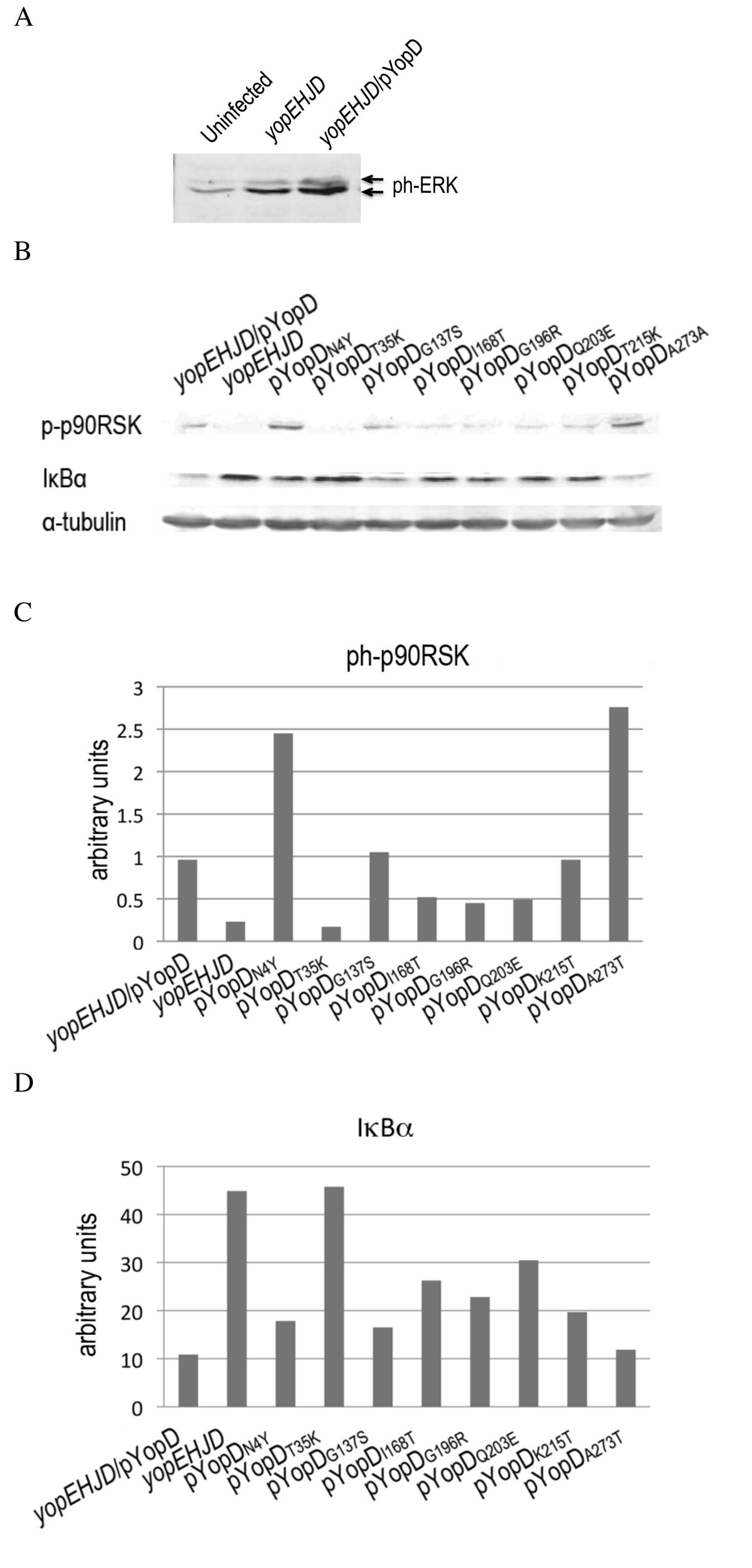

Supplement: S5 Fig — A. HeLa cells were left uninfected or infected with yopEHJD, and yopEHJD/pYopD at MOI of 100 for 1h. Cells were washed, lysed with sample buffer 1X, separated by SDS-PAGE and analyzed by immunoblotting with rabbit anti-phospho ERK. Monoclonal antibody against tubulin was used as a loading control (not shown). B. HeLa cells infected with yopEHJD, yopEHJD/pYopD and yopEHJD expressing the different yopD mutants at the same conditions as described above. Immunoblotting was performed with rabbit anti-phospho p90RSK, anti-IκBα, and anti-tubulin, independently. C and D. Quantification of the signal intensities was performed using Odyssey imaging system software. Values were normalized to tubulin. (TIF) [file pone.0120471.s005.tif]

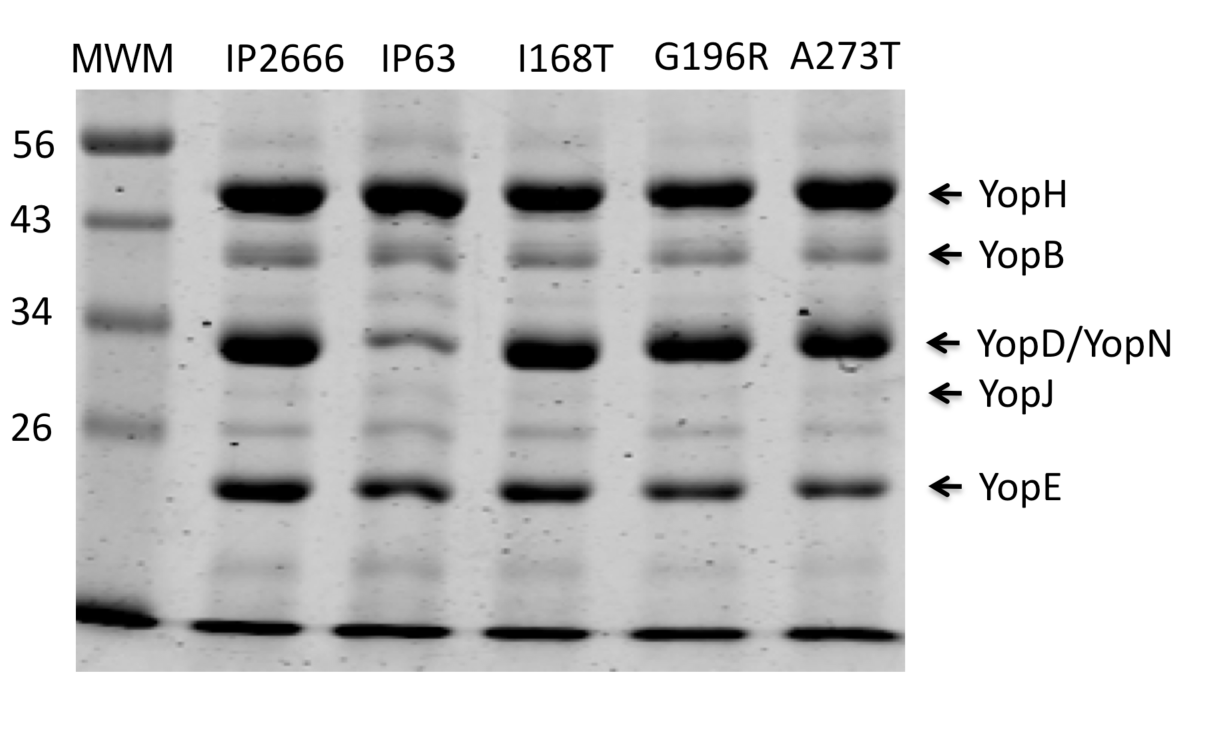

Supplement: S6 Fig — Comassie Blue stained SDS-PAGE gel showing Yop secretion for IP2666 (WT) yopD (IP63) and IP63 endogenously expressing YopDI168T, G196R and A273Y and grown at 37°C at low calcium conditions. (TIF) [file pone.0120471.s006.tif]

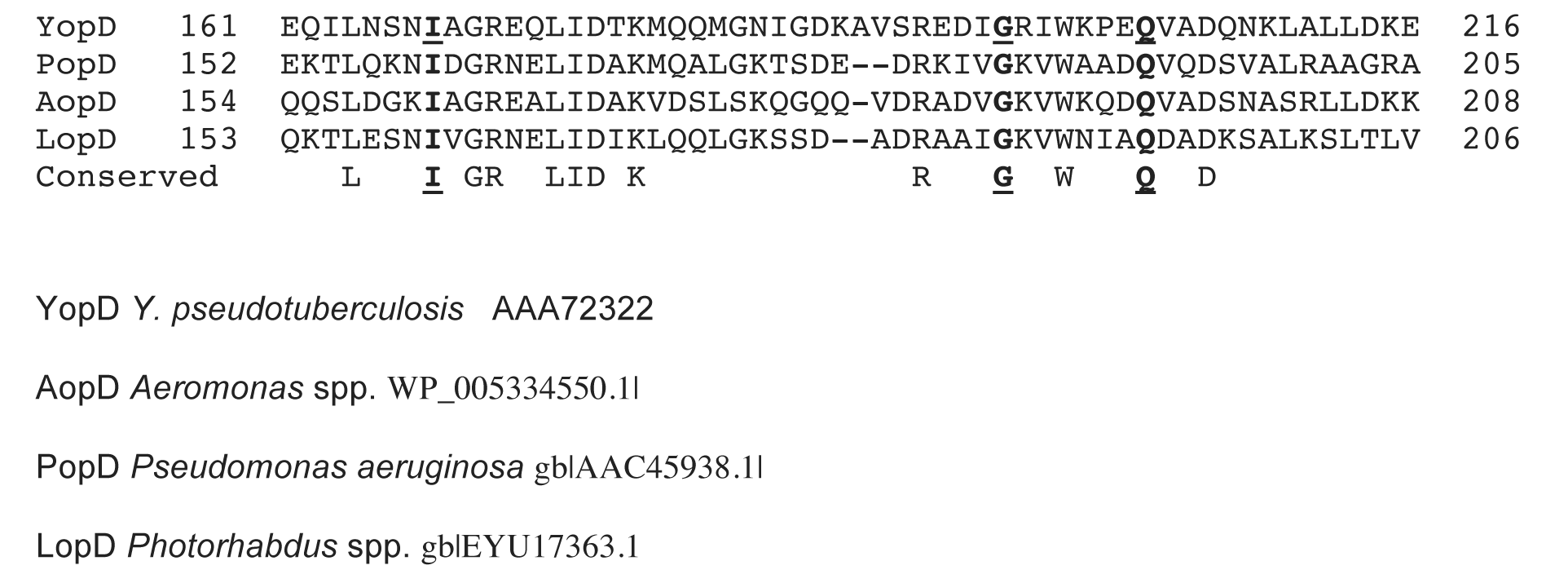

Supplement: S7 Fig — A 55 amino acid region of YopD encompassing residues 168–203, was aligned with the corresponding protein regions of three other YopD homologues: Pseudomonas aeruginosa, Aeromonas spp. and Photorhabdus spp. Identical residues are shown in bold. (TIF) [file pone.0120471.s007.tif]
